# Ambient solid-state triplet–triplet annihilation upconversion in ureasil organic–inorganic hybrid hosts†

**DOI:** 10.1039/d4tc00562g

**Published:** 2024-04-15

**Authors:** Abigail R. Collins, Bolong Zhang, Michael J. Bennison, Rachel C. Evans

**Affiliations:** a Department of Materials Science and Metallurgy, University of Cambridge 27 Charles Babbage Road Cambridge CB3 0FS UK rce26@cam.ac.uk

## Abstract

Triplet–triplet-annihilation upconversion (TTA-UC) has attracted significant attention as an approach to harvest low energy solar photons that cannot be captured by conventional photovoltaic devices. However, device integration requires the design of solid-state TTA-UC materials that combine high upconversion efficiency with long term stability. Herein, we report an efficient solid-state TTA-UC system based on organic–inorganic hybrid polymers known as ureasils as hosts for the archetypal sensitiser/emitter pair of palladium(ii) octaethylporphyrin and diphenylanthracene. The role of the ureasil structure on the TTA-UC performance was probed by varying the branching and molecular weight of the organic precursor to tune the structural, mechanical, and thermal properties. Solid-state green-to-blue UC quantum yields of up to 1.86% were observed under ambient conditions. Notably, depending on the ureasil structure, UC emission could be retained for >70 days without any special treatment, including deoxygenation. Detailed analysis of the structure-function trends revealed that while a low glass transition temperature is required to promote TTA-UC molecular collisions, a higher inorganic content is the primary factor that determines the UC efficiency and stability, due to the inherent oxygen barrier provided by the silica nanodomains.

## Abstract 2

Solid-state green-to-blue upconversion is shown in organic–inorganic hybrid ureasil hosts using the palladium(ii) octaethylporphyrin and diphenylanthracene sensitizer/emitter pair, with emission retained for >70 days without deoxygenation.

## Graphical abstract

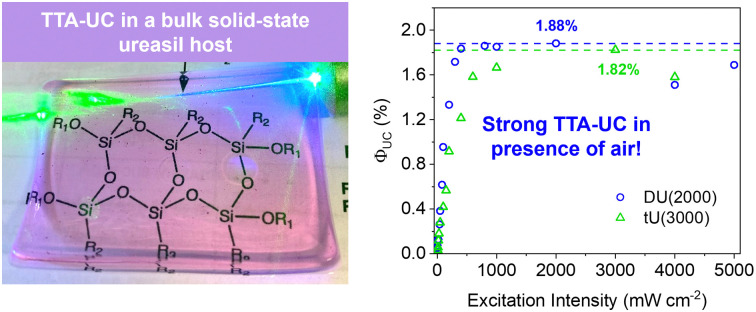

## Introduction

**Fig. 1:**
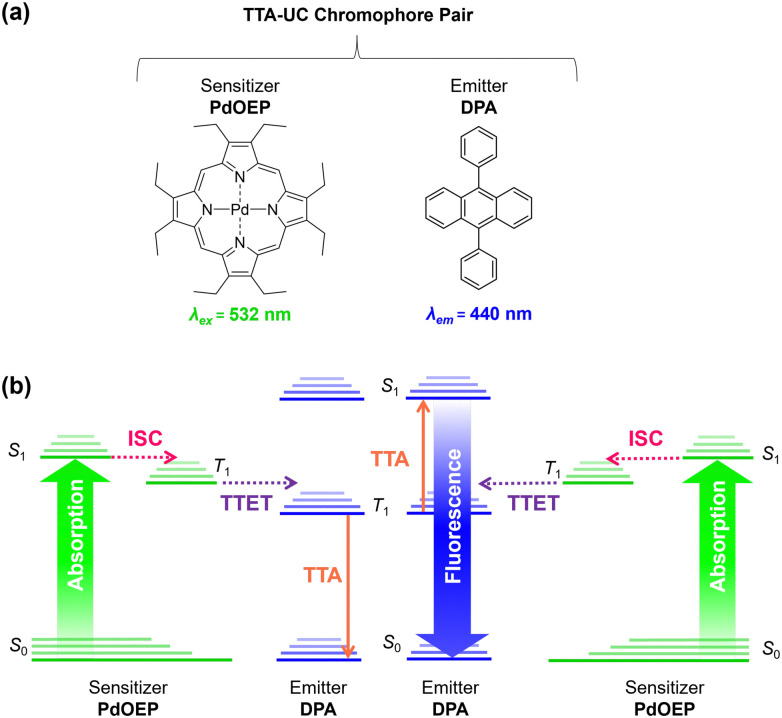
Triplet–triplet annihilation upconversion is a multi-step photophysical process between two chromophores. (a) Structures of the benchmark chromophore pair used in this study: PdOEP (sensitiser) and DPA (emitter). (b) Mechanism of TTA-UC. (i) The absorption of photons by the sensitiser PdOEP (green arrow) results in formation of the excited singlet state (S_1_), which relaxes by intersystem crossing (ISC, pink dashed arrow) to the excited triplet state (T_1_). (ii) Triplet sensitisers then collide with emitters to produce triplet emitters *via* triplet–triplet energy transfer (TTET, purple dashed arrow) if their energy levels overlap within the Dexter radius. (iii) Triplet emitters then collide with each other, leading to triplet–triplet annihilation (orange dashed arrow), whereby one molecule is excited to a higher energy level, and the second relaxes to the ground state. Radiative relaxation of the higher energy state leads to photon emission *via* fluorescence (blue arrow).

Triplet–triplet-annihilation upconversion (TTA-UC) is a photochemical process whereby two low energy photons are converted to a single higher energy photon,^1,2^ and has diverse applications in bioimaging,^3,4^ anti-counterfeiting,^5^ photocatalysis,^6,7^ sensing,^8^ and solar energy harvesting.^9–11^ As illustrated in Fig. 1, TTA-UC occurs between a chromophore pair, comprising a sensitiser with high intersystem crossing (ISC) efficiency, (*e.g.*, palladium(ii) octaethylporphyrin (PdOEP)) and an emitter, such as diphenylanthracene (DPA).^12^ Since TTA-UC requires rapid energy transfer between the sensitiser and emitter, the physical properties of the host medium are also closely linked to the efficiency, quantified by the upconversion quantum yield (*Φ*_UC_, which is capped at 50%).^13,14^ Although high efficiencies (*Φ*_UC_ ∼30%) can be achieved in liquid media,^15–17^ due to rapid molecular collisions and the ease of degassing to prevent oxygen quenching, this has limited practical use. In contrast, a solid-state system will not evaporate or leak and can offer mechanical stability for device integration.

It is typically challenging to achieve a high *Φ*_UC_ in the solid-state,^18,19^ due to the potential problems of parasitic absorption, limited triplet-diffusion, and inhomogeneous chromophore distribution.^20,21^ To overcome these energy sinks, the ideal solid-state host must be transparent and allow good chromophore dissolution and mobility. In addition, it should provide barrier properties to reduce oxygen triplet quenching and a mechanical scaffold for practical integration. These traits are often contradictory and difficult to marry into one system.^19^ As such, a wide variety of materials and architectures have been investigated as solid-state UC hosts including gels,^22–24^ particles,^25–28^ metal–organic frameworks,^29,30^ micro or nanodroplet-containing polymers,^31,32^ and copolymers.^33,34^ Among these, polymer systems are particularly attractive due to their ease of processability and integration with thin film devices. Organic polymers with low glass transition temperature (*T*_g_) such as polyurethanes have been demonstrated to facilitate molecular diffusion, but lack the mechanical stability and oxygen protection that inorganic systems provide.^18,19,35^ In high *T*_g_ polymers such as poly(methyl methacrylate),^36,37^ chromophores are immobilised within the rigid matrix,^38–40^ meaning the triplet–triplet energy transfer (TTET) rate relies entirely on homogenous blending to ensure triplet excitons transfer *via* an energy migration mechanism, leading to lower overall efficiencies.^10,19,20^

**Fig. 2:**
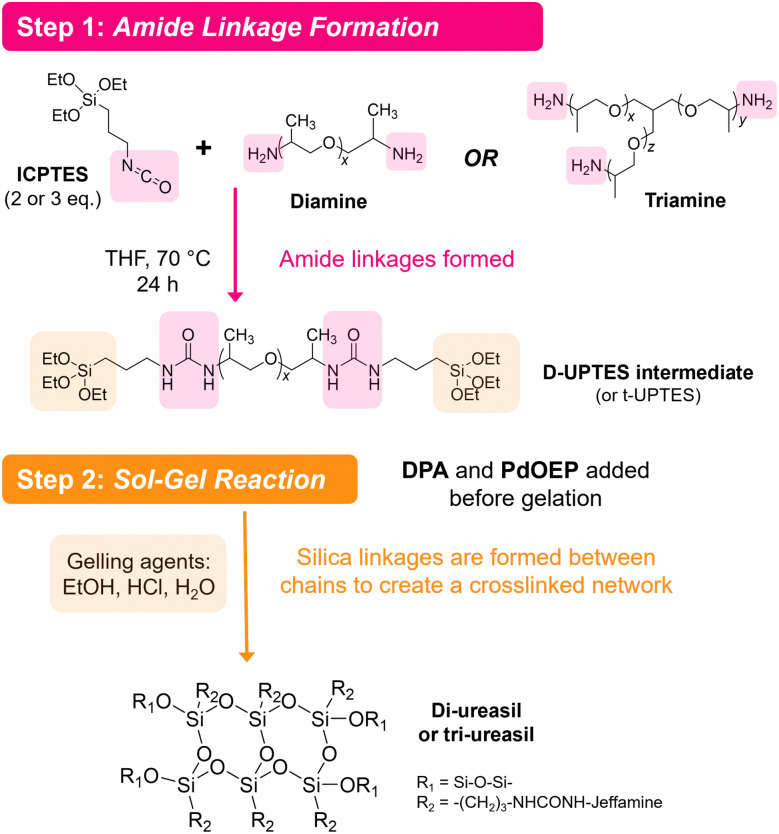
Reaction scheme for solid-state TTA-UC emitters based on ureasils. Two possible polyetheramine precursor branching structures are shown which yield the four ureasils studied - one lower and one higher molecular weight for each. The sensitiser PdOEP and emitter DPA are mixed with the intermediate before sol-gelation.

Organic–inorganic hybrid polymers may offer a solution to these limitations. However, to the best of our knowledge, hybrid polymers have not yet been deployed as TTA-UC hosts, despite offering highly favourable characteristics. For example, ORMOCERS® have been investigated extensively for use as food packaging materials due their excellent barrier abilities against oxygen, water vapor and other compounds.^41–43^ Moreover, with judicious selection of a low *T*_g_ organic precursor, it should be possible to design a hybrid polymer that provides a suitable environment to both solubilise embedded chromophores and facilitate molecular collisions. Herein, we explore this opportunity by demonstrating organic–inorganic hybrid ureasil polymers as chromophore hosts for solid-state TTA-UC for the first time. Ureasils consist of poly(oxyalkylene) chains covalently linked to a siliceous network *via* urea bridges (Fig. 2).^44,45^ The structure and organic-to-inorganic ratio can be easily tuned by altering the branching or molecular weight of the polymer backbone to form monoliths of varying *T*_g_ and flexibility.^46–48^ Ureasils have been used as waveguides for luminescent solar concentrators,^47–49^ electrochromic windows,^50^ and optical communications^46,51^ due to their ability to encapsulate organic and inorganic dopants. Moreover, both non-specific intermolecular forces such as hydrogen bonding, ionic or π–π interactions,^52^ or specific covalent grafting have been demonstrated as successful strategies to both solubilise and localise organic chromophores.^53^

Using the benchmark DPA/PdOEP chromophore pair, we examine the effect the chain length and branching of the organic polymer backbone on the TTA-UC activity in ureasil hosts. While efficient green-to-blue upconversion was observed in all ureasils, the champion host DU(2000) exhibited a maximum *Φ*_UC_ of 1.86 ± 0.05 at 1 W cm^−2^, which is comparable to other similar solid-state systems.^19,54^ Crucially, this efficiency is observed under ambient conditions (*i.e.*, no oxygen removal) and measurable UC persists over 76 days. Moreover, both the UC efficiency and stability can be directly correlated with the organic-to-inorganic ratio in the ureasil host. Through detailed structural analysis, we tentatively link this to faster molecular diffusion for lower *T*_g_ systems, and improved oxygen resistance for those with higher silica content.

## Results and discussion

### Design of ureasil hosts

Ureasils are synthesised using a previously reported two-step process,^46^ in which a poly(oxyalkylene) is first reacted with the silica precursor isocyanatopropyltriethoxysilane (ICPTES) to form urea bridges, before the system is cross-linked at the silicate terminus using sol–gel chemistry (Fig. 2). By varying the length and branching of the poly(oxyalkylene), the ratio of organic to inorganic content in the final ureasil can be tuned. This ratio affects properties such as the *T*_g_ and flexibility of the host. Four poly(oxyalkylene) precursors were investigated and the resulting ureasils are given the following nomenclature according to literature convention: DU(2000), DU(4000), tU(3000), and tU(5000). The *D* or *t* terms refer to the branching, being di- or tri-branched respectively, and the number in brackets is the molecular weight (MW) of the poly(oxyalkylene).

Prior to gelation, ureasils were doped with the sensitiser-emitter pair: PdOEP (0.1 mM with respect to (w.r.t.) the dry monolith) and DPA (10 mM w.r.t. the dry monolith). These ratios were selected based on the UC performance of a set of test samples of tU(3000) loaded with different concentrations and ratios of DPA and PdOEP (see Section S3, Table S2 in ESI†). We note that while high, these sensitiser/emitter concentrations are in line with typical concentrations used in similar solid-state TTA-UC systems.^31,35^ For elevated emitter concentrations, competing relaxation pathways such as self-annihilation and/or reabsorption can occur.^21,55^ As some evidence of these processes was observed at higher (20 mM) emitter concentrations (reduced UC lifetime, decreased UC emission counts, Tables S2 and S3, ESI†), we selected DPA (10 mM) as the upper concentration limit. Reference samples containing just PdOEP or DPA (at equivalent loadings) or no lumophore at all were also prepared for comparison. Once formed, the gels were air-dried to evaporate residual solvent, yielding free-standing monoliths. We note that tri-branched ureasils shrink in size (relative to the initial solution volume) more than di-branched, as do lower molecular weight variants. All ureasils were characterised by powder X-ray diffraction (PXRD) and Fourier transform infrared (FTIR) spectroscopy and display structural features characteristic of ureasils. Full details of the synthetic methods and structural characterisation can be found in the ESI† (ESI,† see Sections S1.2 and S6). It is important to note that all samples were prepared under ambient conditions and not degassed at any point.

**Fig. 3:**
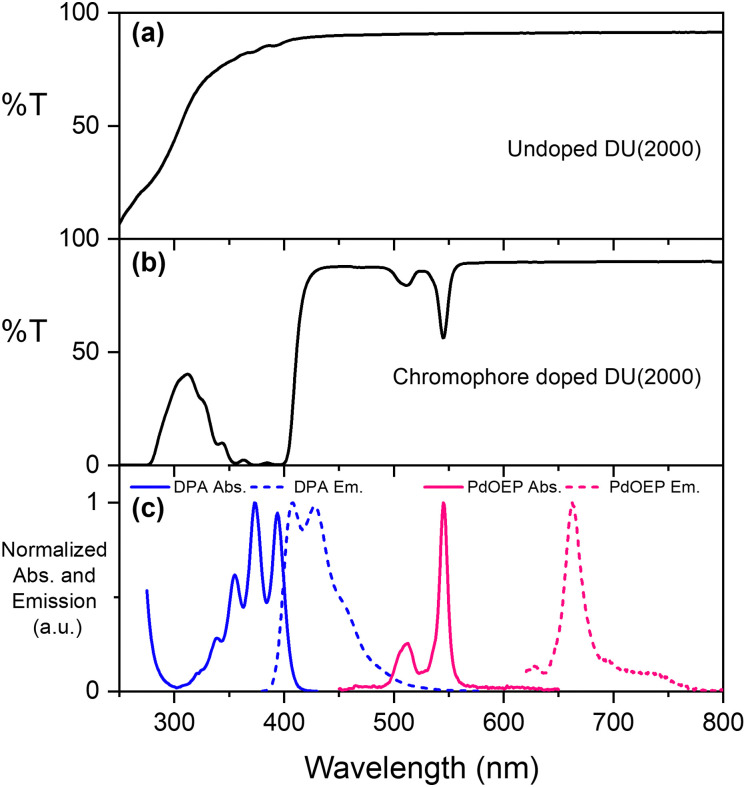
Optical properties of ureasils. (a) % Transmittance (*T*) of parent DU(2000); (b) %*T* of DU(2000) doped with 10 mM DPA and 0.1 mM PdOEP; (c) normalised absorbance (solid lines) and emission (dashed lines) of DPA (blue, *λ*_ex_ = 365 nm, 10^−2^ mM) and PdOEP (pink, *λ*_ex_ = 532 nm, 10^−4^ mM) in THF solution.

The parent ureasils show high optical transmittance (>90%) in the visible range, with a cut-off around 250–300 nm (see Fig. 3a for DU(2000), and Fig. S2 for all other ureasils, ESI†). This cut-off can provide UV-B protection to reduce chromophore photobleaching.^56^ When doped, the ureasils still show high transmittance >600 nm, but as expected absorb strongly in the spectral regions of the chromophores (∼490–560 nm for PdOEP, 300–420 nm for DPA, Fig. 3b). The absorption and emission spectra of DPA (*λ*_ex_ = 375 nm) and PdOEP (*λ*_ex_ = 532 nm) in THF solution are shown in Fig. 3c for reference. Solid-state emission measurements on doped ureasils under selective excitation of either the sensitiser or emitter further confirmed successful integration into the ureasil host (Fig. S4, ESI†).

### TTA-UC activity in ureasils

**Fig. 4:**
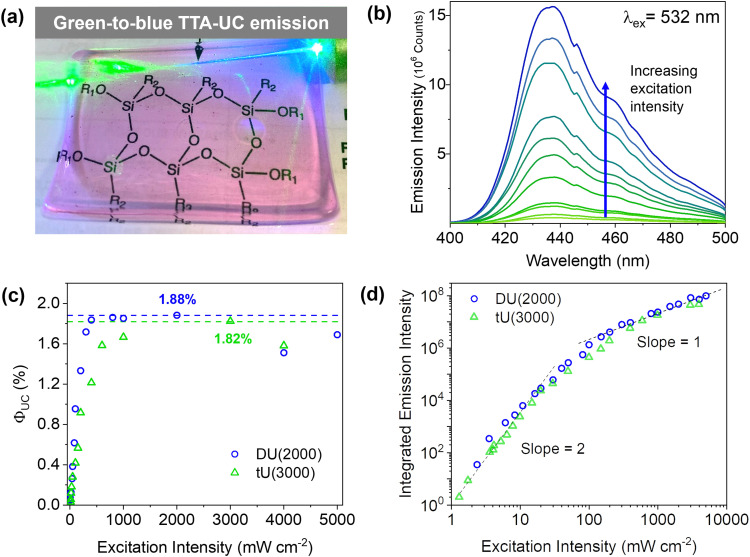
TTA-UC in ureasils. (a) Photograph of chromophore-doped DU(2000) irradiated with a 532 nm laser, which turns visibly blue as it passes through the ureasil. (b) Upconverted DPA emission in DU(2000) with increasing excitation intensity from 8 mW to 5 W cm^−2^. Full-width-half maximum = 40 nm. (c) Upconversion quantum yield of DU(2000) and tU(3000) with increasing excitation intensity. (d) Log–log plot of the excitation intensity dependence of UC emission intensity. The solid lines correspond to linear fits of slopes 2.0 and 1.0 in the low and high excitation intensity regimes.

Doped ureasils show green-to-blue upconversion in ambient conditions when irradiated with a green laser pointer (see Fig. 4a and Fig. S4, Video S1, ESI†). No UC emission is observed in the absence of PdOEP, which supports the sensitised mechanism. These results were confirmed by steady-state photoluminescence spectroscopy (Fig. 4b, and Fig. S5, ESI†). Upon excitation at 532 nm (in the PdOEP absorption band), a broad emission between 400–500 nm is observed, which increases with excitation intensity. We note subtle differences in structure of the UC emission band compared to direct excitation of DPA (at 375 nm in dilute solution, see Fig. 3c), namely the absence of the first vibronic band at 408 nm and the red-shift of the second peak maximum to 440 nm. This behaviour is typical of strong self-absorption due to the high emitter concentration. The photoluminescence quantum yield (*Φ*_PL_) upon direct excitation at 375 nm is >95% for all DPA-ureasils (Table S4, ESI†), which is comparable to the near-unity *Φ*_PL_ of DPA in solution (95% in ethanol).^57,58^ This demonstrates that the ureasil does not affect the performance of DPA as an emitter. In presence of PdOEP, the *Φ*_PL_ (*λ*_ex_ = 375 nm) decreases, but remains high (78–87%, Table 2). This may be due in part to parasitic absorption from the Soret band of the sensitiser (which cannot be isolated from the DPA absorption in the *Φ*_PL_ measurement), along with the presence of new non-radiative relaxation pathways. In both cases, it is noted that the *Φ*_PL_ is higher for DU(2000) and tU(3000) than their higher MW counterparts, and higher in tri-branched than di-branched ureasils. While ureasils are known to emit weakly between 390–600 nm upon excitation at 375 nm (see Fig. S6, ESI†)^47,59^ the *Φ*_PL_ of undoped ureasils (2–7%, with the exception of DU(2000), Table S4, ESI†) is significantly lower than that of DPA. More importantly, no detectable ureasil emission is observed upon excitation at 532 nm, meaning the ureasil host should not offer competing photophysical pathways to the TTA-UC process.

To quantitatively compare the TTA-UC activity in different ureasils, the upconversion quantum yield was measured (see Section S1.5, ESI†). Since the process requires two lower energy photons to emit one photon of higher energy, the maximum *Φ*_UC_ cannot exceed 50%,^18^ and is expressed by:1
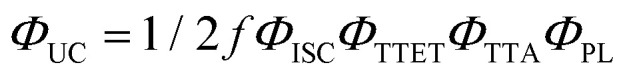
where *Φ*_ISC_, *Φ*_TTET_, *Φ*_TTA_ and *Φ*_PL_ are the quantum yields for ISC, TTET, TTA, and emitter photoluminescence, respectively. The factor *f* represents the probability that a singlet state is produced per TTA step, rather than a triplet or quintet excited state which do not radiatively decay as desired.^60^ Some authors report *Φ*_UC_ as a normalised yield (doubled, up to 100%), however we use the conventional description for reporting *Φ*_UC_, which is capped at 50%.^13^ DU(2000) and tU(3000) both show a *Φ*_UC_ of 1.86 ± 0.05% and 1.67 ± 0.18%, respectively, at a power density of 1 W cm^−2^ of laser excitation at 532 nm. These values are highly efficient compared to similar systems prepared and measured in entirely ambient conditions (*i.e.*, the host system has not been degassed or encapsulated from molecular oxygen).^19^ One of the most efficient of these was a crosslinked epoxy polymer doped with DPA/PtOEP, which was notably prepared and measured in air, achieved a *Φ*_UC_ of 1.9%, 40 mW cm^−2^ threshold intensity, and 17.8 ms UC lifetime.^39^ While higher *Φ*_UC_'s have been achieved in other solid-state media, oxygen removal was employed through use of a physical barrier or addition of scavengers.^35,61,62^

**Fig. 5:**
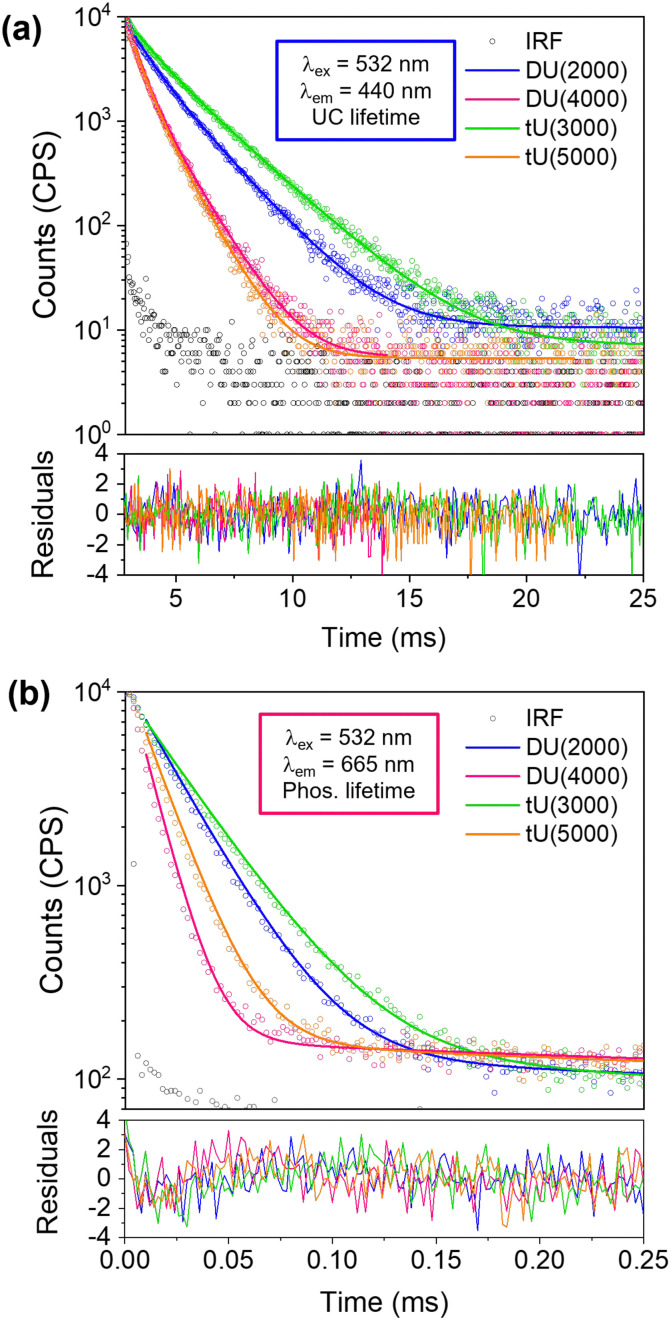
Emission decay studies on TTA-UC ureasils. (a) Upconversion decay curves of PdOEP (0.1 mM)/DPA (10 mM)-doped ureasils DU(2000), DU(4000), tU(3000) and tU(5000) obtained from excitation at 532 nm and detection at 440 nm (with 550 nm short-pass filter). (b) Corresponding phosphorescence decays obtained on excitation at 532 nm and detection at 665 nm (with 550 nm long-pass filter). All measurements were performed under ambient conditions.

**Fig. 6:**
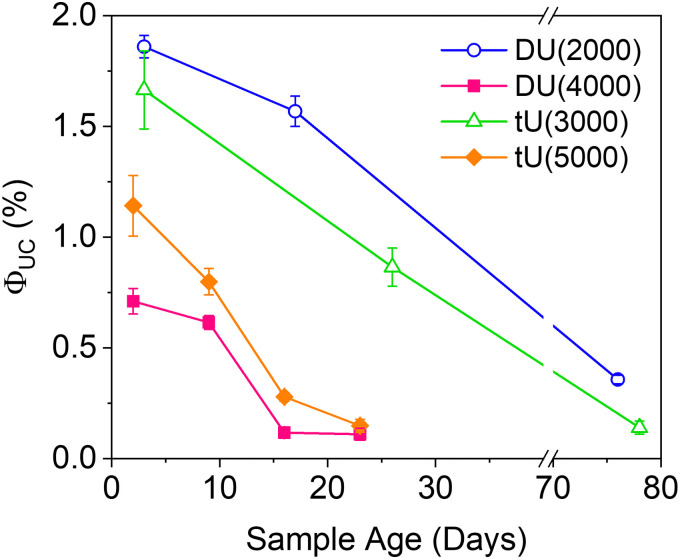
Effect of the ureasil host on the magnitude and stability of the UC quantum yield over time. *λ*_ex_ = 532 nm*, λ*_em_ = 440 nm.

DU(4000) and tU(5000) show lower but still very respectable solid-state *Φ*_UC_'s of 0.71 ± 0.06% and 1.14 ± 0.14%, respectively. This was an unexpected result - structurally, the higher MW ureasils were expected to provide a more liquid-like environment, which should enable fast molecular diffusion for efficient triplet transfer, resulting in a higher *Φ*_UC_, but surprisingly the lower MW ureasils out-perform them. Threshold intensity measurements were carried out on the two leading systems, DU(2000) and tU(3000). The power density threshold (*I*_th_) is the excitation at which the *Φ*_TTA_ is 50% of the maximum value, and the conversion yield is maximised.^63^ The *I*_th_ is a result of a myriad of intrinsic and external factors: a high *Φ*_ISC_, long sensitiser triplet lifetime and efficient TTET will all lower the threshold.^63^ The *I*_th_ of tU(3000) was 27 mW cm^−2^, and UC emission is observed at sub-solar excitation densities as low as 1.5 mW cm^−2^ meaning it can be activated by sunlight (Fig. 5b). Systems with a very low *I*_th_ are highly desired for solar applications as sunlight is a low density light source (100 mW cm^−2^ across solar spectrum, 1.6 mW cm^−2^ at 532  ±  5 nm).^64,65^ DU(2000) had a higher threshold at 166 mW cm^−2^ which could be due its shorter triplet lifetime (Fig. 6), but UC is still seen from the low power density 2.3 mW cm^−2^.

The *Φ*_TTET_ was also evaluated by comparing the phosphorescence intensity of ureasils loaded with only sensitiser only (0.1 mM PdOEP) against those with the sensitiser/emitter pair (0.1 mM PdOEP/10 mM DPA) – see Fig. S8 (ESI†). Lower MW ureasils showed more intense phosphorescence under ambient conditions. This potentially suggests there is increased oxygen protection in this system. Notably all ureasils demonstrate a high *Φ*_TTET_, especially tU(3000) and DU(2000), both with above 95% (Table 1).

**Table 1:** Key photophysical properties of ureasil TTA-UC systems

Ureasil	*Φ*_UC_ a (%)	*I*_th_ b (mW cm^−2^)	*Φ*_PL_ d (%)	*Φ*_TTET_ e (%)	〈*τ*_UC_〉^f^ (ms)	〈*τ*_PHOS_〉^g^ (with DPA) (μs)	〈*τ*_PHOS_〉^g^ (no DPA) (μs)
DU(2000)	1.86 ± 0.05	166	81.4 ± 2.1	97 ± 2	16 ± 0.5	102	25
DU(4000)	0.71 ± 0.06	—c	78.4 ± 0.8	84 ± 3	10 ± 0.3	327	9
tU(3000)	1.67 ± 0.18	27	86.6 ± 2.0	95 ± 2	22 ± 2.5	59	25
tU(5000)	1.14 ± 0.14	—c	83.9 ± 1.3	92 ± 3	8 ± 0.6	182	8

a Upconversion quantum yield at excitation intensity of 1 W cm^−2^ (532 nm).

b Threshold intensity determined from logarithmic plot of integrated UC emission intensity at varying 532 nm laser power.

c Not measured.

d Photoluminescence quantum yield of the emitter determined by the integrating sphere method (*λ*_ex_ = 375 nm).

e TTET calculated from average phosphorescence emission intensity with and without emitter present (see eqn S3 (ESI), *λ*_ex_ = 532 nm, *λ*_em_ = 600–800 nm).

f Average upconversion lifetime (*λ*_ex_ = 532 nm, *λ*_em_ = 440 nm).

g Average phosphorescence lifetime in absence or presence of DPA (*λ*_ex_ = 532 nm, *λ*_em_ = 665 nm). All measurements performed under ambient conditions. Errors are the standard deviation of two measurements.

Due to the bimolecular nature of TTA, the rate of TTA will show a quadratic dependence on the concentration of excited triplet emitters (and thus excitation intensity). In diffusion-controlled ideal solutions, a clear distinction between low (quadratic) and high (linear) excitation intensity regimes can often be discerned.^21^ However, in (semi)solid-state systems, a more gradual shift between the two regimes is more commonly observed,^21,66^ as is the case here (Fig. 4d). Transition to linear dependence with excitation intensity implies that the concentration of triplet excited emitters is sufficient to ensure a high probability of annihilation collisions within their excited state lifetime, leading to near-unity triplet relaxation by TTA. However, in solid-state systems, sensitiser/emitter pairs are under non-ideal diffusion-control: the mean free path of both chromophores is likely to be more restricted compared to fluid solution, which may lead to less random collisions. As such, the change in slope from linear to quadratic may be less well-defined, as idealised TTET behaviour competes with alternative relaxation processes such as sensitiser self-annihilation.^67^

### Lifetime studies

We had expected the lower *T*_g_'s of the higher MW organic precursors (Table 2) would lead to a higher rate of molecular diffusion and therefore improved UC performance; yet we see the opposite trend. To explore why this occurs, we next investigated the UC decay kinetics. The UC emission is very long-lived (milliseconds) and display a rise-time that cannot easily be reconvoluted from the instrument response function (see Fig. S9, ESI†). As such, the decay lifetimes were determined by tail fits (Fig. 5a).^67^ Full details of the fitting parameters are available in Table S5 (ESI†). Two exponential decay components were required to fit the decays: a shorter-lived, minor component (*f*_1_ = 18–34%, *τ*_1_ = 5–11 ms) attributed to partially oxygen-quenched UC emission, and a longer-lived component attributed to the natural UC emission (*f*_2_ = 66–82%, *τ*_2_ = 11–22 ms). The average upconversion lifetime 〈*τ*_UC_〉 follows the trend tU3000 > DU2000 > tU5000 = DU4000, which mirrors the calculated inorganic silica content (Table 2 and Table S4, ESI†). 〈*τ*_UC_〉 decreases with increasing polymer host MW and reduced branching. Previous studies on poly(ethylene)-poly(ethylene glycol)-SiO_2_ block copolymers observed that the oxygen transfer rate (OTR) was reduced when the ratio of inorganic component was increased.^68^ Moreover, polymers with a lower MW organic component showed a lower OTR due to reduced chain mobility.^69^ It is therefore highly probable that these combined factors also determine the observed trends in the ureasil system. We note that while it has been reported that DPA can form an endoperoxide bridge with singlet molecular oxygen, providing a certain degree of intrinsic oxygen protection,^70^ this cannot explain the differences that arise here since the DPA concentration is identical in all ureasils under investigation.

**Table 2:** Physical properties of undoped ureasil samples

Ureasil	Silica content^a^ (wt%)	Coherence length, *L*^b^ (Å)	Structural unit distance, *d*^b^ (Å)	Shrinkage^c^ (%)	*T*_g_ (°C)	Elastic modulus (MPa)	Bending modulus (MPa)
DU(2000)	4.1	17.28	4.30	55.5	−70	1.16 ± 0.002	4.93 ± 0.20
DU(4000)	2.1	15.55	4.34	50.3	−73	0.53 ± 0.001	1.95 ± 0.21
tU(3000)	4.1	17.39	4.28	49.7	−64	1.90 ± 0.004	9.39 ± 0.25
tU(5000)	2.5	15.64	4.35	40.8	−82	0.97 ± 0.002	3.67 ± 0.66

a Determined from the molecular weight of inorganic silica with respect to the MW of the ureasil (see ESI Section S1.2 and Table S1).

b From PXRD (see ESI, Section S5.3).

c Determined from the volume of the sol before casting and the final area of the solid ureasil when dried (see ESI, Section S1.2).

The phosphorescence decays in air were also measured for sensitiser/emitter and sensitiser-only ureasils (Fig. 5b and Fig. S9b, Table S6 (ESI†), PdOEP = 0.1 mM in both systems). This system is unusual as the lifetime with the emitter present is longer – the opposite of the usual case. This is thought to be due to back-transfer of triplet from the DPA emitter extending the lifetime of phosphorescence.^71^ The shorter lifetimes in PdOEP doped ureasils could also be due to relatively high concentrations of PdOEP leading to self-annihilation due to TTA between excited PdOEP triplets at high excitation intensities.^67^

### Long-term UC stability

Air-stability is a huge challenge in the field of TTA-UC and the demand for materials with inherent oxygen protection is high. However, the long term *Φ*_UC_ over time in air is seldom reported. Fig. 6 shows the fluctuation in *Φ*_UC_ for samples measured over nearly 3 months. Notably, DU(2000) and tU(3000) retained their activity over a significantly longer period (up to 76–78 days) than the samples with the lower %wt silica content. In contrast, *Φ*_UC_ for DU(4000) and tU(5000) dropped off rapidly and was negligible after 24 days. Air-stability decreases with increasing polymer host molecular weight and reduced branching.

For all samples, the loss in efficiency is likely due to two factors: atmospheric oxygen ingress into the ureasil leading to triplet quenching, and photobleaching of the chromophores over time. Fig. S10 (ESI†) shows the UV/Vis absorption spectra over time (up to 23 days post gelation). While the PdOEP absorption remains effectively constant in all ureasils, the DPA absorption decreases with time, suggesting photobleaching may be the primary factor. We note that ureasils themselves are typically photostable, with accelerated aging studies using intense UV irradiation (24 hours) leading to no change in optical performance.^48^

### Effect of host structure on TTA-UC performance

Our photophysical studies demonstrate that the relationship between the ureasil host structure and the TTA-UC performance is non-trivial. If we consider the di- or tri-branched series individually, a lower MW and higher silica content leads to ureasils with higher *Φ*_UC_ and increased stability. However, if we compare analogues between the two series, the trends are less clear.

**Fig. 7:**
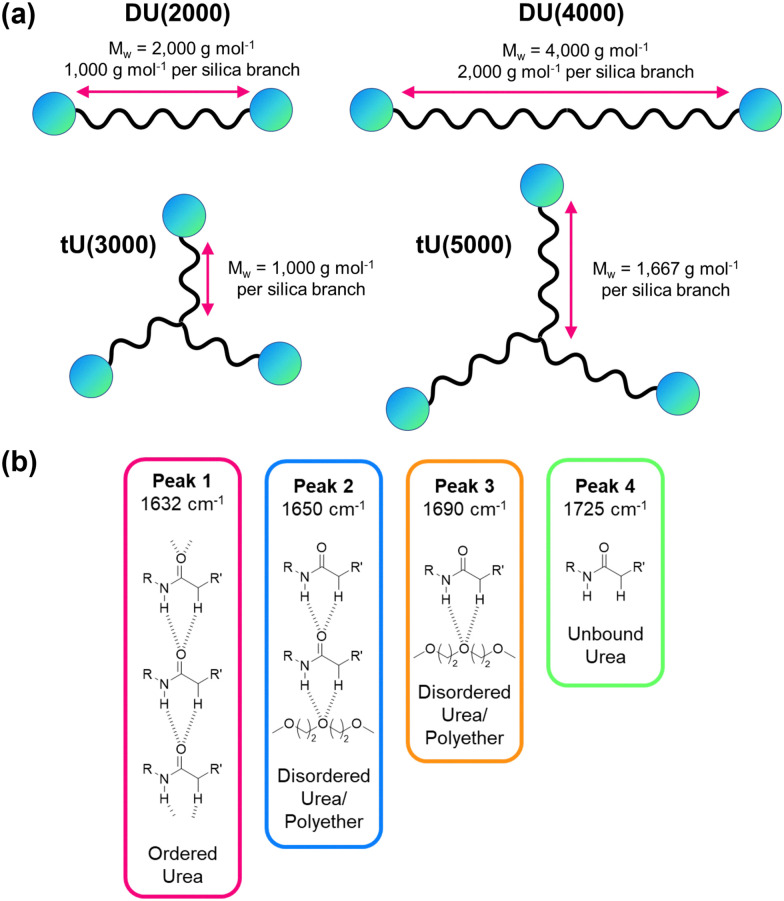
Structural features of ureasil hosts. (a) Schematic representation of the effect of changing the MW and branching of the polymer precursor on the organic-to-inorganic ratio within the overall ureasil structure. The wavy black line represents organic polymer, and the blue circles represent inorganic silica, which is then crosslinked. (b) Hydrogen-bonding associations and corresponding peak assignments in the amide I region of the FTIR spectra of ureasils. *R* and *R*′ represent the inorganic and organic portion of the structure, respectively.

Table 2 summarises the key structural, thermal, and mechanical properties of the undoped ureasils. All ureasils have a *T*_g_ well below 0 °C, as determined by dynamic mechanical analysis (DMA), indicating that at room temperature the polymer backbone should be in a viscous liquid or rubbery state with individual polymer chains moving freely (see also Fig. S12 and Table S7, ESI†). DMA measures viscoelasticity and gives a direct order of viscosity between ureasil samples, which is used as a proxy for chromophore diffusion rates. This below-ambient *T*_g_ suggests that all ureasils investigated should offer good chromophore solubility and a reasonable degree of mobility to facilitate the essential molecular collisions that for TTET. For both series, increasing the precursor chain length leads to a decrease in *T*_g_, which is attributed to both an increase in chain flexibility^68^ and a decrease in the number of urea-silica linkages. This effect is more significant for the tri-branched series, for which the incidence of cross-linkages is inherently higher (Fig. 7a).

Further insight into the local organic structure can be obtained by examining the FTIR spectra in the amide I region (1600–1800 cm^−1^), which reveals the extent of hydrogen bonding (see Fig. 7b, Fig. S12 and Table S8, ESI†).^44^ Ureasils with a lower precursor MW have a lower proportion of free unbound urea groups (Peak 4, Fig. 7b and Fig. S12, ESI†) and weak, disordered hydrogen bonds (Peak 3, Fig. 7, and Fig. S12, ESI†) and a higher fraction of direct, ordered hydrogen- bonding from N–H to C=O on neighbouring chains (Peak 1). A higher contribution from ordered hydrogen-bonding peaks is linked with a higher *T*_g_ and more closely packed structure.^44,47^ Moreover, increased levels of hydrogen-bonding order have also been shown to decrease oxygen penetration in related organic–inorganic polymers.^69^

DU(2000) and tU(3000) showed the highest *Φ*_UC_ – these samples also have the lower MW chain for the di-and tri-branched series, respectively. However, tri-branched samples will contain a more condensed silica network than di-branched samples of comparable MW. The silica content (wt%) in the sample is therefore a good indirect measure of the combined effects of branching and chain length. For both di- and tri-branched precursors, the lower MW leads to a higher silica content (both *ca.* 4.1%), due to the shorter distance between chain ends as illustrated schematically in Fig. 7a. This can be observed at the macroscopic level: ureasils with the lowest wt% silica are more flexible and bendy (Fig. S13, ESI†), and therefore have higher elastic and bending moduli (see Table 2, and Fig. S13, S14, ESI†). Similarly, tri-branched structures have higher bending moduli than their di-branched counterparts.

PXRD provides insight into the size and coherence of the silica domains and the distance between them (see Fig. S15 and Table S9, ESI†) at the sub-nanometer scale.^53^ Interestingly, while the structural unit distance is consistent across all samples (*d* ∼4.3 Å, from Bragg's law), the coherence length, *L*, over which the structural unit survives (*i.e.* the crystallite size) is longer for both DU(2000) and tU(3000). This is somewhat counterintuitive, since a more persistent and condensed silica network would be expected to reduce lumophore mobility. This suggests that the molecular collisions responsible for TTA-UC are primarily localised to the organic domains (which is reasonable based on solubility grounds), and that as long as the *T*_g_ is below room temperature, there will be sufficient mobility for these to occur.

To examine the effect of oxygen exclusion on the UC performance, a preliminary test was conducted on DPA (10 mM)/PdOEP (0.1 mM)-tU(3000) in which the precursor solution was degassed by nitrogen bubbling for 1 hr before gelation. A slight increase in 〈*τ*_UC_〉 and large increase in 〈*τ*_PHOS_〉 was observed (to 289 μs, Table S10, ESI†), and *Φ*_UC_ increased to 2.39 ± 0.30% at 1 W cm^−2^. This indicates that while the ureasil structure provides some intrinsic oxygen barrier (as shown by the long stability), higher TTET rates and *Φ*_UC_ could possibly be achieved with additional oxygen removal.

## Conclusions

We have demonstrated ureasils as highly effective host materials for solid-state TTA-UC, achieving *Φ*_UC_ up to 1.86% (at 1 W cm^−2^ excitation density) in ambient conditions. The UC efficiency varies with the ureasil host used. All ureasils studied inherit high optical transparency, mechanical strength, and oxygen protection from the inorganic silica nanodomains, whilst retaining the chromophore solubility, low *T*_g_, flexibility and processability of organic polymers. However, it is the balance between the organic-to-inorganic ratio and the persistence length of the silica domains that determines both the absolute efficiency and long-term stability of the UC emission. Of the four ureasils studied, DU(2000) and tU(3000) contain the highest silica content and domain coherence length. We postulate that these structural features help provide increased oxygen protection, as shown by the enhanced triplet lifetime and *Φ*_UC_. Impressively, measurable UC emission is retained for both samples for >70 days without any special treatment or storage, including deoxygenation. This ability to operate in ambient conditions due to inherent oxygen protection is highly desirable for almost all TTA-UC applications. We are currently undertaking detailed studies of the O_2_ transmission rates and local diffusivity in these ureasils and structurally-related analogues to fully identify the key structure-function characteristics. The underpinning knowledge obtained through this investigation will inform the design of next generation hybrid host structures, with the goal of further enhancing the efficiency and long-term stability of TTA-UC in the solid-state.

## Conflicts of interest

There are no conflicts to declare.

## Supplementary Material

TC-012-D4TC00562G-s001

TC-012-D4TC00562G-s002
