# Supplementary material for: Ambient solid-state triplet–triplet annihilation upconversion in ureasil organic–inorganic hybrid hosts
Source: J Mater Chem C Mater. 2024 Apr 15;12(17):6310–8. doi: 10.1039/d4tc00562g (PMC11064974; doi:10.1039/d4tc00562g)
Supplement: TC-012-D4TC00562G-s001 [file TC-012-D4TC00562G-s001.pdf]

Electronic Supplementary Material (ESI) for Journal of Materials Chemistry C.
This journal is © The Royal Society of Chemistry 2024

Supporting Information

Ambient Solid-State Triplet-Triplet Annihilation Upconversion in Ureasil Organic-Inorganic Hybrid Hosts

Abigail R. Collins[a], Bolong Zhang[a §], Michael J. Bennison[a], and Rachel C. Evans[a]*

[a] Department of Materials Science & Metallurgy, University of Cambridge, 27 Charles Babbage Road, CB3 0FS, U.K.

* Corresponding Author: rce26@cam.ac.uk

§ Present address: Key Laboratory of Functional Materials and Devices for Special Environments of CAS, Xinjiang Key Laboratory of Electronic Information Materials and Devices, Xinjiang Technical Institute of Physics & Chemistry of CAS, Urumqi, 830011, China

TABLE OF CONTENTS

1. Experimental Methods 3

1.1. Materials 3

1.2. Fabrication of organic-inorganic hybrid ureasils 3

1.3. Ultraviolet/visible transmittance spectroscopy 5

1.4. Steady-state photoluminescence (PL) spectroscopy 5

1.5. Upconversion (UC) quantum yield 5

1.6. Time-resolved emission measurements 7

1.7. Fourier transform infrared (FTIR) spectroscopy 8

1.8. Mechanical testing 8

1.9. Dynamic mechanical analysis (DMA) 9

1.10. Powder X-ray diffraction (PXRD) 9

2. Optical properties of parent ureasil hosts 9

3. Optimization of sensitizer/emitter ratio 9

4. TTA-UC activity in ureasils 13

4.1. Calculation of TTET efficiency 16

4. Lifetime studies 16

5. Long-term UC stability 20

6. Effect of host structure on TTA-UC performance 21

6.1. Thermal characterization 21

6.2. FTIR analysis 22

6.3. Mechanical characterization of ureasils 23

6.4. PXRD: determination of structural unit distance and coherence length 24

7. References 27

1. Experimental Methods

1.1. Materials

Tetrahydrofuran (THF, ≥99.9%), ethanol (95.0%), hydrochloric acid (37%) and 3-(triethoxysilyl)propylisocyanate (ICPTES, 95.0%) were purchased from Fisher Scientific. 9,10-Diphenylanthracene (DPA, 99%) was purchased from Alfa Aesar. 2,3,7,8,12,13,17,18-Octaethyl-21*H*,23*H*-porphine palladium (II) (PdOEP, 85%) was purchased from Sigma Aldrich. Water was obtained from a Millipore Simpak 2 water purification system. All materials were used as received.

JEFFAMINE® D-X and t-X (*D* denotes *bis*-end functionality, *t* denotes *tris*-end functionality, and *X* is representative of the molecular weight, MW): poly(propylene glycol) b*is*(2-aminopropyl ether) (JEFFAMINE® D-2000, MW = 2000 g mol^{-1} JEFFAMINE® D-4000, MW = 4000 g mol^{-1}) were purchased from Merck; glyceryl poly(oxypropylene) triamine (JEFFAMINE® T-3000, MW = 3000 g mol^{-1}, JEFFAMINE® T-5000, MW = 5000 g mol^{-1}) were purchased from Huntsman.

1.2. Fabrication of organic-inorganic hybrid ureasils

Ureasils were prepared via a two-step sol–gel process. ICPTES is mixed with JEFFAMINE® in a molar ratio of 2:1 for *bis*-functionalised amines and 3:1 for *tris*-functionalised amines in THF. The reaction mixture was refluxed at 70 °C for 24 h to obtain the organic–inorganic hybrid precursor, diureapropyltriethoxysilane (d-UPTES) or triureapropyltriethoxysilane (T-UPTES) in solution. In the second step, gelling reagents were sequentially added (ethanol, HCl (0.5 M) and water) to either d-UPTES or T-UPTES and thoroughly mixed. The molar ratio of ICPTES:ethanol:HCl:water used was 176:350:1:265. For chromophore doped ureasils, stock solutions of DPA and PdOEP were dissolved in THF and added to the mixture before the gelling reagents were added. The resulting mixture was poured into a polypropylene mould and gelled into free-standing monoliths via the sol-gel process. The mould was sealed with Parafilm M® to ensure slow evaporation of excess THF in the samples over 1-2 days, followed by further oven drying at 40°C for 1-2 days, until the excess THF had evaporated.

The shrinkage of each ureasil was calculated by the original volume of solution before gelation and the final volume of the solid-state gel host after THF evaporation is complete. The shrinkage is calculated and then used to reverse engineer the desired final concentration of DPA and PdOEP in the dry gel. For example, if the ureasil shrinks by 50%, the original solution should be 50% diluted in chromophore concentration before gelation.

The weight percentage of silica was calculated according to Table S1, based on the molecular weight of O-Si-O linkages over the molecular weight of the entire polymer chain and urea linkage. The calculation assumes that the monolith is completely dry and gives an indication of the ratio of organic to inorganic domains and their subsequent contribution to the material properties.

Table S1. Calculation of silica weight percentage (wt%) for organic-inorganic polymer hybrids.

Ureasil	Linkages	Repeat units	MW of PEO[a]	MW of chain[b]	MW of chain / linkage[c]	MW of silica[d]	Silica wt%
**DU(2000)**	2	33	58	2056	1028	44	4.1
**DU(4000)**	2	68	58	4086	2043	44	2.1
**tU(3000)**	3	50	58	3083	1027.67	44	4.1
**tU(5000)**	3	85	58	5113	1704.33	44	2.5

[a] PEO = polyethylene oxide in JEFFAMINE precursor. [b] Calculated MW of the total organic polymer chain (including organic amide links formed in step 1 of synthesis). [c] MW of organic polymer divided by the number of linkages for each chain (2 for di-ureasils, 3 for tri-ureasils). [d] Molecular weight of inorganic -O-Si-O- linker.

1.3. Ultraviolet/visible transmittance spectroscopy

UV/Vis transmittance spectra were measured with a PerkinElmer Lambda 750 spectrophotometer using wavelength scan with a resolution of 1 nm at a scan speed of 267 nm/min and a slit width of 2 nm. Solid-state samples (thickness = 1 mm) were directly mounted to the sample holder.

1.4. Steady-state photoluminescence (PL) spectroscopy

Steady-state PL spectroscopy was performed on a Fluorolog-3 spectrophotometer (Horiba Jobin Yvon). Solid-state emission spectra were recorded using the front-face configuration. The excitation and emission slits were adjusted so that the maximum PL intensity was within the range of linear response of the detector and were kept the same between samples if direct comparison between the emission intensity was required. Emission and excitation spectra were corrected for the wavelength response of the system and the intensity of the lamp profile over the excitation range, respectively, using correction factors supplied by the manufacturer. Photoluminescence quantum yields (PLQYs) were measured using a Quanta-phi integrating sphere (Horiba Jobin Yvon) mounted on the Fluorolog-3 spectrophotometer.

1.5. Upconversion (UC) quantum yield

The UC emission spectra, phosphorescence spectra, threshold intensity (I_{th}), UC quantum yield (UCQY) and UC fluorescence lifetime of all samples were measured with an FLS 1000 TCSPC spectrometer (Edinburgh Instruments Ltd.). Samples were excited with a 532 nm laser (MGL-III-532, 200mW), with a short-pass filter (cut-off 500 nm, Thorlabs) applied in front of the detector. The laser power was adjusted using Thorlabs PM100A Power Meter Console combined with a S120VC Si photodiode power sensor (range: 200-1100 nm).

The UCQY was measured with an integrating sphere (SNS125 5-inch sphere, three windows, International Light Technologies, Figure S1). The sample was cut into a 1 cm^2 square and loaded at the center of the sphere by a sample holder. A baffle is placed in front of the observation window, which blocks any scattering and reflection of the laser from the sample surface. The angle of the sample holder is adjustable. The normal direction of the sample holder is 22.5° to the excitation beam line, which leads the reflection of the laser to the inner surface of the sphere.

The laser power was measured with a photodiode before each UCQY measurement. Both the emission of the sample (380-500 nm) and scattering of the laser beam (530-534 nm) were measured. A neutral density filter (O.D.=3.0) was placed before the excitation beam for the scattering intensity measurements. Six data sets were collected to calculate the UCQY of each sample: 1. sample in the path of the beam – "in fluorescence"; 2. sample in scattering; 3. sample facing away from beam – "out of fluorescence", 4. sample out of scattering; 5. empty sphere fluorescence; 6. empty sphere scattering.

Three sets of data were obtained for three 1 cm^2 sections of ureasil sample. The parallel data sets were calculated separately, which gives three UCQY results for each sample, and the reported UCQY is the average of these data, along with the standard deviation of the measurements. During the 'sample in beam' measurement, the sample was facing toward both the excitation window and the observation window, while in the sample-out mode, the holder was turned 180° to have the back of the holder facing the windows. For the fluorescence measurement (Data sets 1 and 3), the bandwidth was 1 nm for the detector, and the scan step was 1 nm per data point with duration of 1 second, scanned from 380 nm to 500 nm. For the scattering measurement (Data sets 2 and 4), the bandwidth was 1 nm for the detector, and the scan step was 0.1 nm per data point with duration of 0.1 second, scanned from 530 nm to 534 nm. The transmittance of the filter at the excitation wavelength was measured with a UV-Vis absorption spectrometer (DS5, Edinburgh Instruments Ltd.), taking the average over 10 parallel measurements. The empty-sphere data sets (Data sets 5 and 6) were collected at the beginning of the measurement, under the same conditions of the sample-in measurement, which were shared in all calculations of samples measured in the same day. During the calculation, all data were corrected by the transmittance of each filter used, and normalized based on the slit-width, scan step and the scan duration used.

The UCQY was calculated using the experimental approach described by Porrès *et al.*[1] and the following formulae:[2,3]

$$\Phi_{\mathrm{UC}} = \frac{E_{x,in} - (1 - A)E_{x,out}}{AL_{b,in}} \qquad \text{(Eq. S1)}$$

where A is the percentage of the photons absorbed directly by the sample, which is corrected by removing the secondary absorption from the sphere-reflected photons:

$$A = \frac{L_{x,out} - L_{x,in}}{L_{x,out}} \qquad \text{(Eq. S2)}$$

where E is the integrated photon counts from emission spectra, and L is the integrated photon counts from the scattering spectra. x delineates sample, while b is blank. *In* means the sample was in the path of the excitation beam, and *out* delineates the sample is out of the beam line. A quantum yield is defined as the ratio of absorbed to emitted photons, meaning the UCQY is limited to 50% since this is a bimolecular process. While some papers report this as a normalised value, UCQY is reported to its un-normalised value here, to a maximum of 50%.[4]

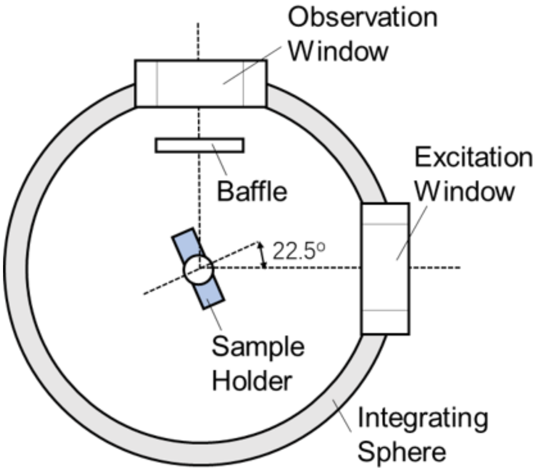

Figure S1. Integrating sphere set-up used to determine UCQYs.

1.6. Time-resolved emission measurements

Fluorescence decay measurements were performed using the multi-channel scaling (MCS) method on a FLS1000 PL spectrometer. The emission decay was recorded using a high-speed photomultiplier tube (PMT-980) equipped with TCC2 counting electronics. The UC fluorescence decay profile was measured using 532 nm laser excitation (MGL-III-532 laser) and a visible PMT-980 detector, with a short-pass filter (cut-off 500 nm, Thorlabs) applied in front of the detector. The plus repetition rate was 12.5 kHz (80 ms), the laser power was set to its maximum, and the pulse width was gradually increased until more than 1000 counts/s was observed at 440 nm. For phosphorescence lifetimes, a long-pass 550 nm filter was used in place of the 500 nm cut off filter and the repetition rate increased to1 kHz (1 ms). The instrument response function (IRF) was measured using with a SiO_2 particle suspension solution (Ludox® colloidal silica) using a neutral density filter (OD=3) in front of the excitation source, and with no emission filter.

Individual tail-fits were applied to each emission decay curve using the FAST software package (Edinburgh Instruments) using a multiexponential decay function:

$$I(t) = \sum_{i}^{n} \alpha_i e^{-t/\tau_i} \qquad \text{(Eq. S3)}$$

where α_i and τ_i are the amplitude and lifetime of the ith component, respectively, and α_i is normalized to unity for n components. The goodness of fit was assessed using the reduced chi-square statistics, χ^2, and the randomness of the residuals.[5] To facilitate comparison of the emission decay behaviour between different samples, the average lifetime $\langle\tau\rangle$ and fractional contributions (f_i) are also reported, which for a bi-exponential decay is given by:

$$\langle\tau\rangle = \frac{\alpha_1\tau_1^2 + \alpha_2\tau_2^2}{\alpha_1\tau_1 + \alpha_2\tau_2} = f_1\tau_1 + f_2\tau_2 \qquad \text{(Eq. S4)}$$

where

$$f_i = \frac{\alpha_i\tau_i}{\sum_j^n \alpha_j\tau_j} \qquad \text{(Eq. S5)}$$

1.7. Fourier transform infrared (FTIR) spectroscopy

FTIR spectra were obtained using a Nicolet iS5 (ThermoFisher instruments) spectrometer fitted with an attenuated total reflectance (ATR) diamond press at room temperature over a range of 4000-400 cm^{-1} with a resolution of 0.5 cm^{-1}, averaging 64 scans. Deconvolution of the Amide I region (1800-1600 cm^{-1}) was performed using a non-linear least square (NLLS) fit to a Gaussian function in Origin 2019 (OriginLab) software. The quality of all fits was assessed qualitatively by comparison of the cumulative fit peak (given by the sum of the intensity of each of the fitted peak at a given x value) to the raw data.

1.8. Mechanical testing

4-Point flexural testing and uniaxial tensile testing were performed on a Tinius Olsen 1ST using a 25 N load cell. Samples were cut into tensile specimens using a HPC Laser Ltd. Laserscript LS3060 to produce a 15 mm × 3 mm testing area with a 4.5 mm gripping area at the ends. In the 4-point geometry, samples were placed on supports 12 mm apart and loaded from above at a speed of 1 mm/min by two loading points separated by 4 mm. The force and corresponding displacement on these loading points was recorded until the point at which the loading points were compressing rather than bending the sample. In the uniaxial tensile geometry, samples were gripped at both ends and pulled apart at a speed of 2 mm/min until failure was observed. The force and corresponding displacement of the grips was recorded until failure. The datasets were analysed in Origin 2019 (OriginLab) to generate the elastic and flexural moduli of the samples from the gradient of the initial straight region of the stress-strain (first 100 data points) or force-extension curves (first 500 data points), respectively. In the case of the 4-point geometry, the experimental data was first transformed by multiplying the force required for deflection by a sample-dependent constant, k, and plotted against the

position of the grips. This constant was determined from $k = \left(\frac{5a^3}{6I}\right)$, where *a* is the distance between loading points (4 mm) and *I* is the second moment of area of the sample about the bending neutral axis. *I* was calculated from $I = \frac{bh^3}{12}$, where *b* is the width of the sample and *h* is the sample thickness. Repeats were taken of each sample.

1.9. Dynamic mechanical analysis (DMA)

Dynamic mechanical analysis was performed on a TA Instruments Q800 DMA in 4-point bending mode over the temperature range -100–300°C at a heating rate of 5 °C/min. The samples were cut to dimensions 50 mm × 8 mm × 1-2 mm to fit the double cantilever.

1.10. Powder X-ray diffraction (PXRD)

PXRD was performed on a B3 Bruker D8 Advance. The samples were exposed to the Cu Kα radiation (λ = 1.54 Å) at room temperature and measured using silicon substrate to minimise background scattering. The investigated 2θ range was 0°–50°.

2. Optical properties of parent ureasil hosts

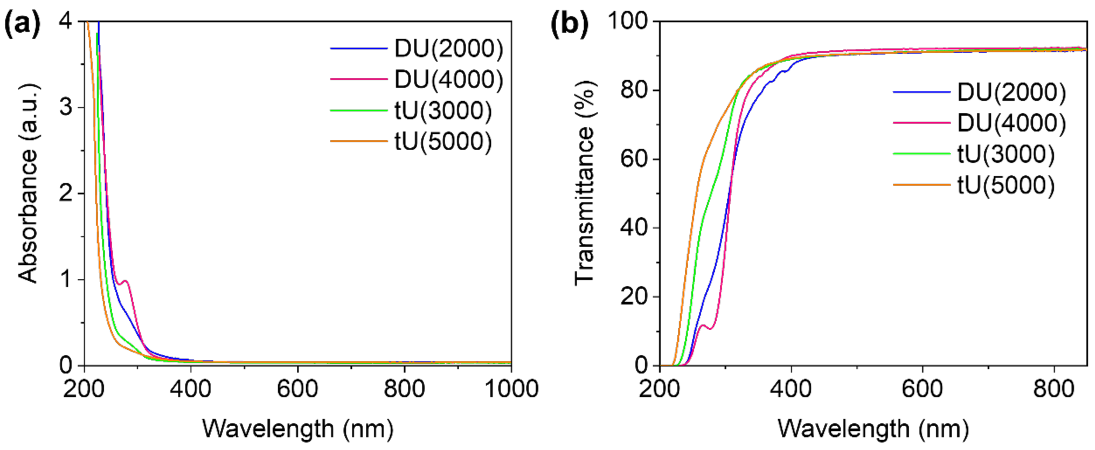

Figure S2. Optical spectra of undoped ureasils (1 mm thickness). (a) Absorption spectra and (b) % transmittance spectra.

3. Optimization of sensitizer/emitter ratio

The effect of sensitizer/emitter (S/E) concentration was investigated in the tU(3000) host to optimize the TTA-UC performance based on the upconversion quantum yield (Φ_{UC}) and upconversion lifetime (τ_{UC}). The results are summarized in Table S2. It was found that doubling the concentration of both chromophores produced the same TTA-UC efficiency within error. The effect of doubling or halving the concentrations of S/E was also investigated and, in both cases, showed that doubling/halving the ratio between them lowered the TTA-UC

efficiency from 16% to 12%. When DPA concentration was doubled to 20 mM, the TTA-UC efficiency rose to 20±5% from 16%, but the total number of UC emission counts decreased, which suggests that self-annihilation may be occurring.

Analysis of the upconversion lifetime data (Figure S3, Table S3) confirmed these trends. All decay curves required a bi-exponential fit to the data, with the quality of fit evaluated by the evenness of the residuals. The intensity-averaged upconversion lifetime was found to be longest at an S:E ratio of 1:100, as seen in the 10 mM DPA/0.1 mM PdOEP and 20 mM DPA/0.2 mM PdOEP systems. These also have a larger contribution from the first lifetime component (τ_1) than other samples. Doubling or halving the emitter concentration leads to a reduction in the average UC lifetime. The 20 mM DPA/0.1 mM PdOEP system had a high UCQY but a reduced lifetime compared to 10 mM DPA/0.1 mM PdOEP system. This is likely due to increased self-annihilation at elevated emitter concentrations. Shorter lifetimes were also observed when doubling or halving the sensitizer concentration. Based on these observations, we selected the DPA (10 mM) and PdOEP (0.1 mM) as the emitter/sensitizer concentration to be used in all samples.

Table S2. Investigation of optimum chromophore concentrations in ureasil tU(3000). Rough measurements were taken of key parameters to determine UC performance.

DPA conc. (mM)	PdOEP conc. (mM)	Φ_{PL} (%)[a]	UC intensity ($\times 10^7$ CPS)[b]	Φ_{UC}(%)[c]	$\langle\tau_{UC}\rangle$ (ms)[d]
5	0.1	71.5 ± 0.7	1.8 ± 0.1	1.22 ± 0.26	33.6
20	0.1	85.4 ± 0.3	2.1 ± 0.2	2.04 ± 0.50	34.8
10	0.05	90.1 ± 1.4	1.1 ± 0.2	**	20.1
10	0.2	67.8 ± 1.1	4.6 ± 0.3	1.26 ± 0.28	21.6
10	0.1	87.2 ± 1.2	2.5 ± 0.4	1.64 ± 0.53	28.7
20	0.2	69.6 ± 1.8	6.1 ± 0.7	1.62 ± 0.12	24.3

[a] Photoluminescence quantum yield of the emitter determined by the integrating sphere method (λ_{ex} = 375 nm). [c] Upconversion quantum yield at excitation intensity of 1 W cm^{-2} (532 nm). [c] Upconversion emission intensity is the integrated area under UC emission (380-500 nm). [d] Average upconversion lifetime (λ_{ex} = 532 nm, λ_{em} = 440 nm) determined from a bi-exponential fit. ** Insufficient absorbance by PdOEP at this concentration to measure accurately.

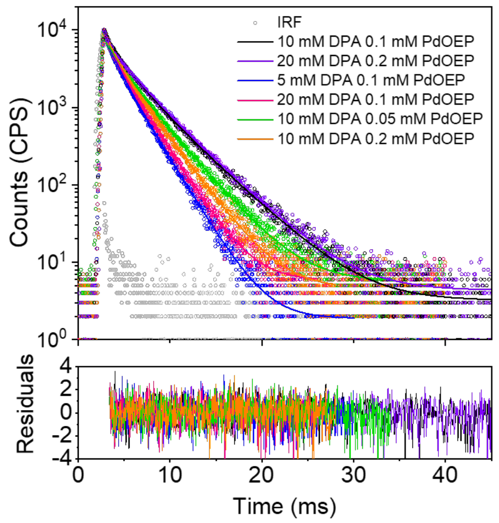

Figure S3. Upconversion decay curves (open symbols), fits (solid lines) and residuals of PdOEP/DPA-doped ureasil tU(3000) at varied concentrations, obtained from excitation at 532 nm and detection at 440 nm (with 550 nm short-pass filter). All fits were obtained via tail-fitting to a bi-exponential function.

Table S3. Summary of fitting parameters obtained for double exponential tail fits to the upconversion lifetime decay curves (Figure S3) at different sensitizer/ emitter concentrations. λ_{ex} = 532 nm, λ_{em} = 440 nm.

Concentration	χ^2 [a]	Component	α_i [b]	f_i (%)[c]	τ_i (ms)[c]	<τ_{UC}> (ms)[d]
10 mM DPA 0.1 mM PdOEP	1.479	1	0.47 ± 0.02	29 ± 1	18	33.6
		2	0.53 ± 0.02	71 ± 3	40	
20 mM DPA 0.2 mM PdOEP	1.474	1	0.36 ± 0.02	21 ± 2	19	34.8
		2	0.64 ± 0.03	79 ± 4	39	
5 mM DPA 0.1 mM PdOEP	1.334	1	0.29 ± 0.02	16 ± 1	10	20.1
		2	0.71 ± 0.02	84 ± 3	22	
20 mM DPA 0.1 mM PdOEP	1.398	1	0.23 ± 0.02	11 ± 1	9.9	21.6
		2	0.77 ± 0.02	89 ± 2	23	
10 mM DPA 0.05 mM PdOEP	1.383	1	0.34 ± 0.01	17 ± 1	13	28.7
		2	0.66 ± 0.01	83 ± 2	32	
10 mM DPA 0.2 mM PdOEP	1.353	1	0.33 ± 0.01	17 ± 1	11	24.3
		2	0.67 ± 0.01	83 ± 2	27	

[a] Chi-squared, a measure of goodness of fit. [b] Normalized pre-exponential factor for *i*th component. [c] Fractional contribution of the ith component. [d]Lifetime of the *i*th component. [d] Average emission lifetime.

4. TTA-UC activity in ureasils

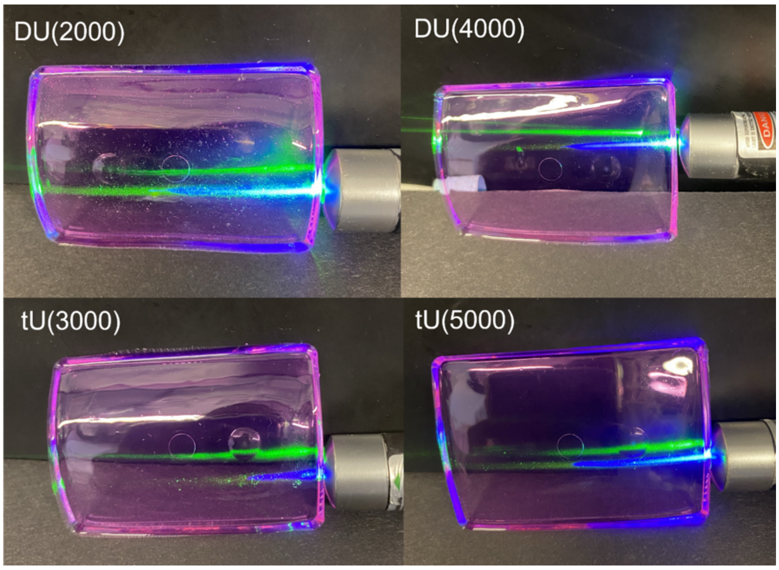

Figure S4. Photographs of all four doped ureasils exhibiting green-to-blue upconversion in ambient conditions under excitation with a 532 nm laser pointer.

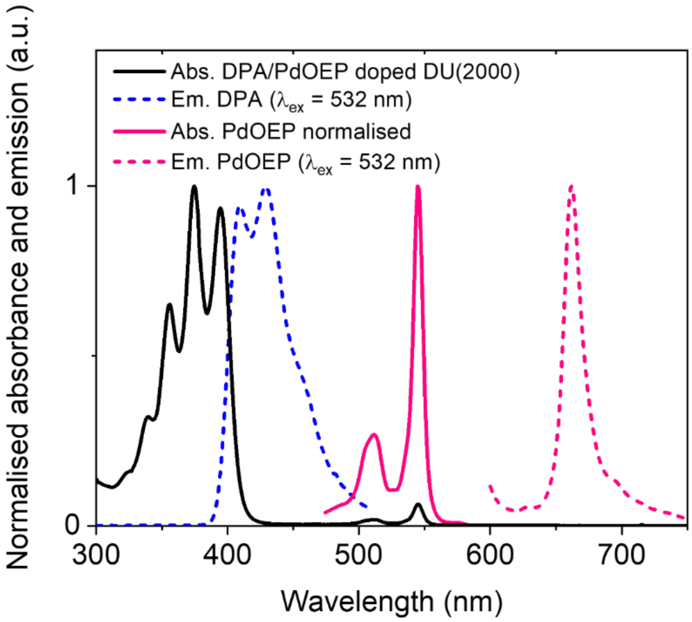

Figure S5. Optical properties of (0.1 mM)/DPA (10 mM) in the solid-state DU(2000) host. The absorption (solid black line, normalised to DPA maximum) and emission spectra (normalised to peak maximum for each component) for DPA (dashed blue lines) and PdOEP (dashed pink lines) obtained upon excitation at 532 nm are shown. The normalised PdOEP absorption band for this sample (solid pink lines) is also included to facilitate comparison.

Table S4. Photoluminescence quantum yields (Φ_{PL}) of emitter-doped and sensitizer/emitter-doped ureasils.

Sample	Chromophore[a]	Φ_{PL}
DU(2000)	–[b]	31.9 ± 3.2
	10 mM DPA	100.3 ± 1.5
	10 mM DPA/0.1 mM PdOEP	81.4 ± 2.1
DU(4000)	–[b]	5.2 ± 0.5
	10 mM DPA	98.7 ± 0.3
	10 mM DPA/0.1 mM PdOEP	78.4 ± 0.8
tU(3000)	–[b]	2.3 ± 0.4
	10 mM DPA	98.9 ± 2.4
	10 mM DPA/0.1 mM PdOEP	86.6 ± 2.0
tU(5000)	–[b]	6.7 ± 0.8
	10 mM DPA	95.1 ± 0.5
	10 mM DPA/0.1 mM PdOEP	83.9 ± 1.3

[a] λ_{ex} = 375 nm, measured emission range λ_{em} = 380-530 nm. [b] Intrinsic ureasil emission. We note that the Φ_{PL} was negligible (non-measurable) at λ_{ex} = 532 nm for undoped ureasils.

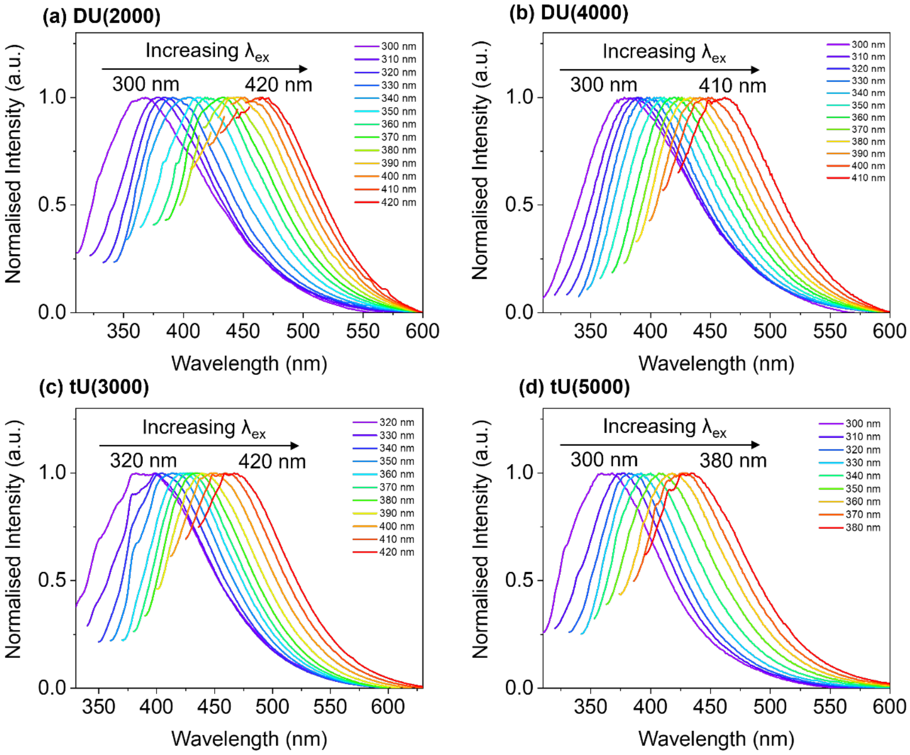

Figure S6. Normalized emission spectra of undoped ureasil samples as a function of excitation wavelength. The emission bands can be resolved into two components: a longer-wavelength blue component is ascribed to the donor-acceptor electron-hole recombination at the urea linkages *via* proton transfer, and the short-wavelength purple-band band is due to localized oxygen defects at the siliceous nanodomains.[6,7]

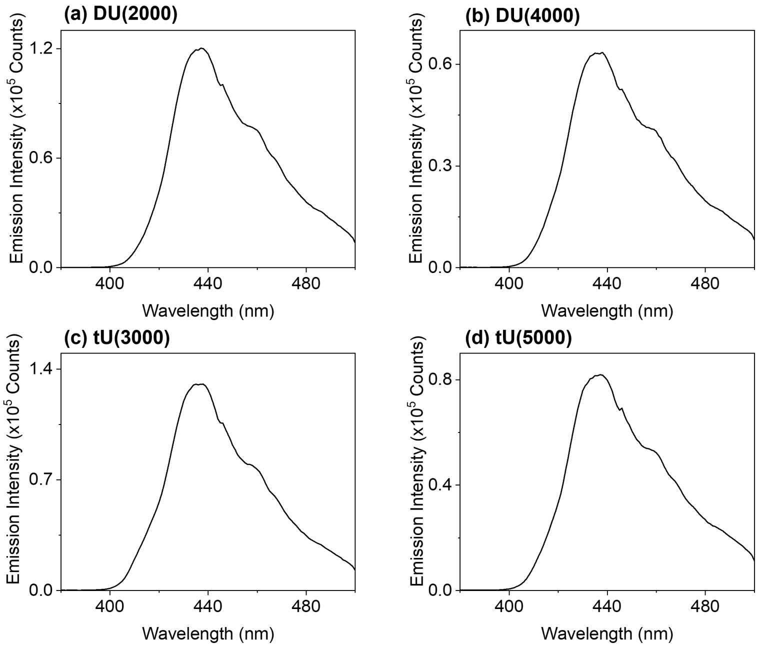

Figure S7. Upconversion emission spectra of all DPA/PdOEP doped ureasils excited with a 532 nm laser at 1000 mW cm^{-2}. These spectra are from one measurement and serve as a demonstration of the UC emission spectrum. Repeat measurements were taken to yield the average UCQY and associated error.

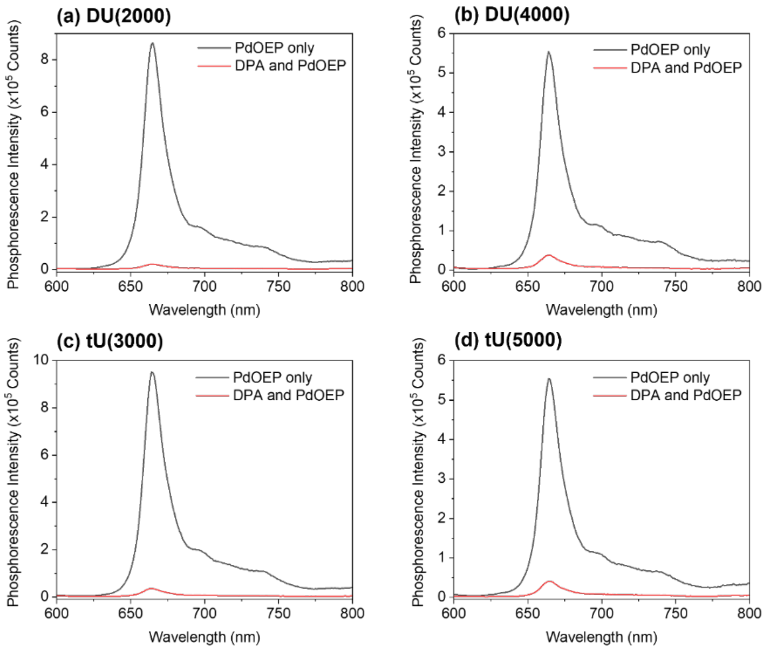

Figure S8. Phosphorescence emission spectra of all ureasils (λ_{ex} = 532 nm at 1,000 mW cm^{-2}, λ_{em} = 600-800 nm) doped with 0.1 mM PdOEP only (black lines) and doped with 10 mM DPA and 0.1 mM PdOEP (red lines). These spectra are from one measurement and serve as a demonstration of the emission spectrum. Repeat measurements were taken to yield the average TTET and associated error.

4.1. Calculation of TTET efficiency

$$\Phi_{\mathrm{TTET}} = 1 - \frac{integrated\ phosphorescence\ emission\ (sensitizer\ and\ emitter)}{integrated\ phosphorescence\ emission\ (sensitizer\ only)} \quad \text{(Eq. S6)}$$

Equation S5 was used to calculate the TTET efficiency from the phosphorescence spectra of ureasil samples doped with just the sensitizer or the sensitizer/emitter pair. An average of three emission scans were taken for each doped ureasil to obtain an error from the standard deviation.

4. Lifetime studies

Table S5. Summary of fitting parameters obtained for double exponential tail fits to the upconversion lifetime decay curves. λ_{ex} = 532 nm, λ_{em} = 440 nm.

**Ureasil**	χ^{2} [a]	**Component**	α_i [b]	f_i [c]	τ_i **(ms)** [d]
DU(2000)	1.253	1	0.37 ± 0.02	20 ± 1	7.9 ± 0.3
		2	0.63 ± 0.02	80 ± 3	18 ± 0.7
DU(4000)	1.292	1	0.52 ± 0.03	31 ± 2	5.0 ± 0.1
		2	0.48 ± 0.03	69 ± 5	12 ± 0.7
tU(3000)	1.294	1	0.32 ± 0.02	18 ± 1	11 ± 1.4
		2	0.68 ± 0.02	82 ± 3	22 ± 3.5
tU(5000)	1.255	1	0.52 ± 0.02	34 ± 2	5.0 ± 0.6
		2	0.48 ± 0.03	66 ± 4	11 ± 1.4

[a] Chi-squared, a measure of goodness of fit. [b] Normalized pre-exponential factor for *i*th component. [c] Fractional contribution of the ith component. [d] Lifetime of the *i*th component.

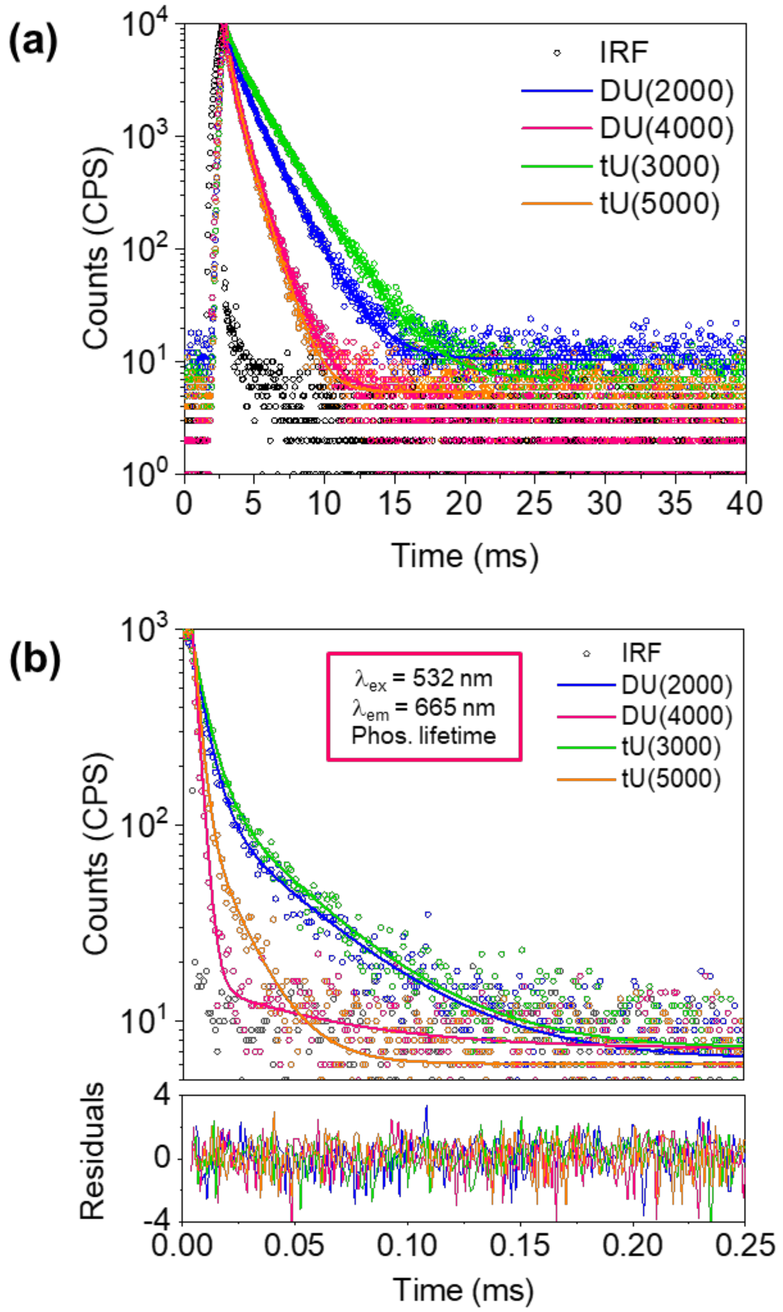

Figure S9. Decay curves of DPA/PdOEP doped ureasils. (a) Upconversion decay curves shown in Figure 5a with scale adjusted to show the rise in emission prior to decay. (b) Phosphorescence decay curves (open symbols), fits (solid lines) and residuals for ureasil samples doped with sensitizer only (0.1 mM PdOEP). Measurement conditions: 532 nm laser excitation, detecting at 665 nm until1,000 counts, at 1 kHz with a 550 nm long-pass filter.

Table S6a. Summary of fitting parameters obtained for double exponential tail fits to the phosphorescence decay curves for DPA (10 mM)/PdOEP(0.1 mM)-doped ureasils. λ_{ex} = 532 nm, λ_{em} = 665 nm.

**Ureasil**	$\chi^{2\,a}$	**Component**	α_i ^{b}	f_i **(%)**^{c}	τ_i **(μs)**^{c}

DU(2000)	1.229	1	0.991 ± 0.0057	83 ± 0.5	22
		2	0.009 ± 0.0003	17 ± 1	490 ± 40
DU(4000)	1.259	1	0.983 ± 0.011	48 ± 0.5	10
		2	0.017 ± 0.0004	52 ± 1	620
tU(3000)	1.210	1	0.989 ± 0.052	89 ± 0.5	28 ± 2
		2	0.011 ± 0.0007	11 ± 1	310 ± 60
tU(5000)	1.236	1	0.987 ± 0.008	67 ± 0.5	15
		2	0.013 ± 0.0003	33 ± 1	520

[a] Chi-squared, a measure of goodness of fit. [b] Normalized pre-exponential factor for *i*th component. [c] Fractional contribution of the ith component. [d] Lifetime of the *i*th component.

Table S6b. Summary of fitting parameters obtained for double-exponential tail fits to the phosphorescence decay curves for PdOEP(0.1 mM)-doped ureasils (sensitizer only). λ_{ex} = 532 nm, λ_{em} = 665 nm.

**Ureasil**	$\chi^{2\,a}$	**Component**	α_i [b]	f_i **(%)**[c]	τ_i **(μs)**[d]
DU(2000)	1.232	1	0.84 ± 0.02	43 ± 1	5.6 ± 0.7
		2	0.16 ± 0.01	57 ± 3	40 ± 5
DU(4000)	1.349	1	0.99 ± 0.03	88 ± 2	2.5
		2	0.006 ± 0.001	12 ± 2	56
tU(3000)	1.310	1	0.81 ± 0.02	42 ± 1	6.2 ± 0.2
		2	0.19 ± 0.01	58 ± 3	38 ± 2.6
tU(5000)	1.385	1	0.89 ± 0.03	59 ± 2	2.7
		2	0.11 ± 0.01	41 ± 4	15

[a] Chi-squared, a measure of goodness of fit. [b] Normalized pre-exponential factor for *i*th component. [c] Fractional contribution of the ith component. [d] Lifetime of the *i*th component.

5. Long-term UC stability

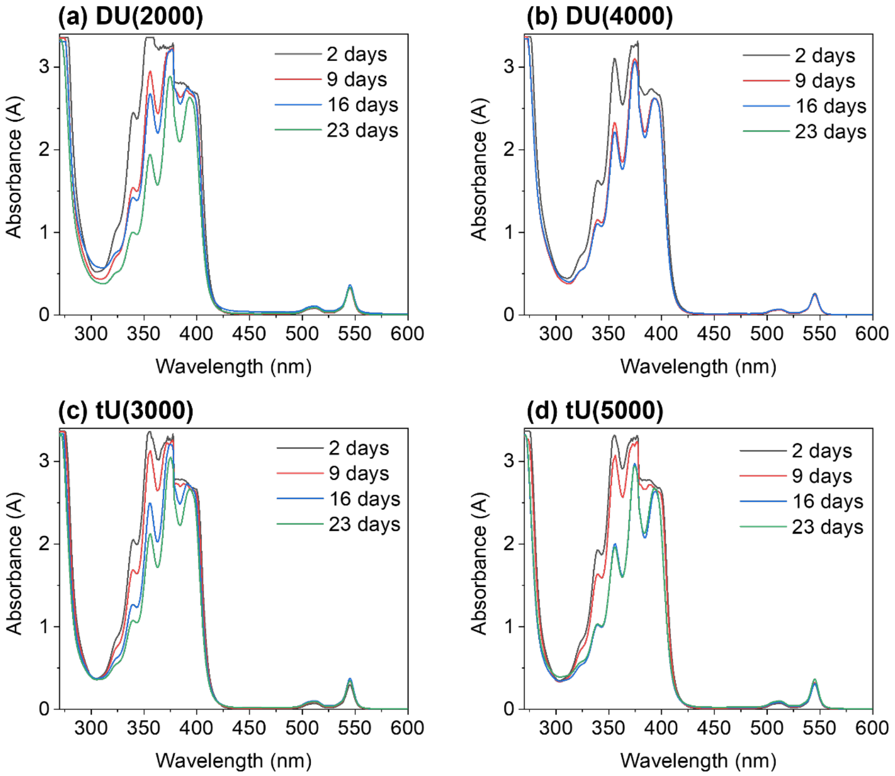

Figure S10. UV-Vis absorption spectra of DPA/PdOEP doped ureasils at increasing time intervals after gelation showing the photodegradation of DPA over time.

6. Effect of host structure on TTA-UC performance

6.1. Thermal characterization

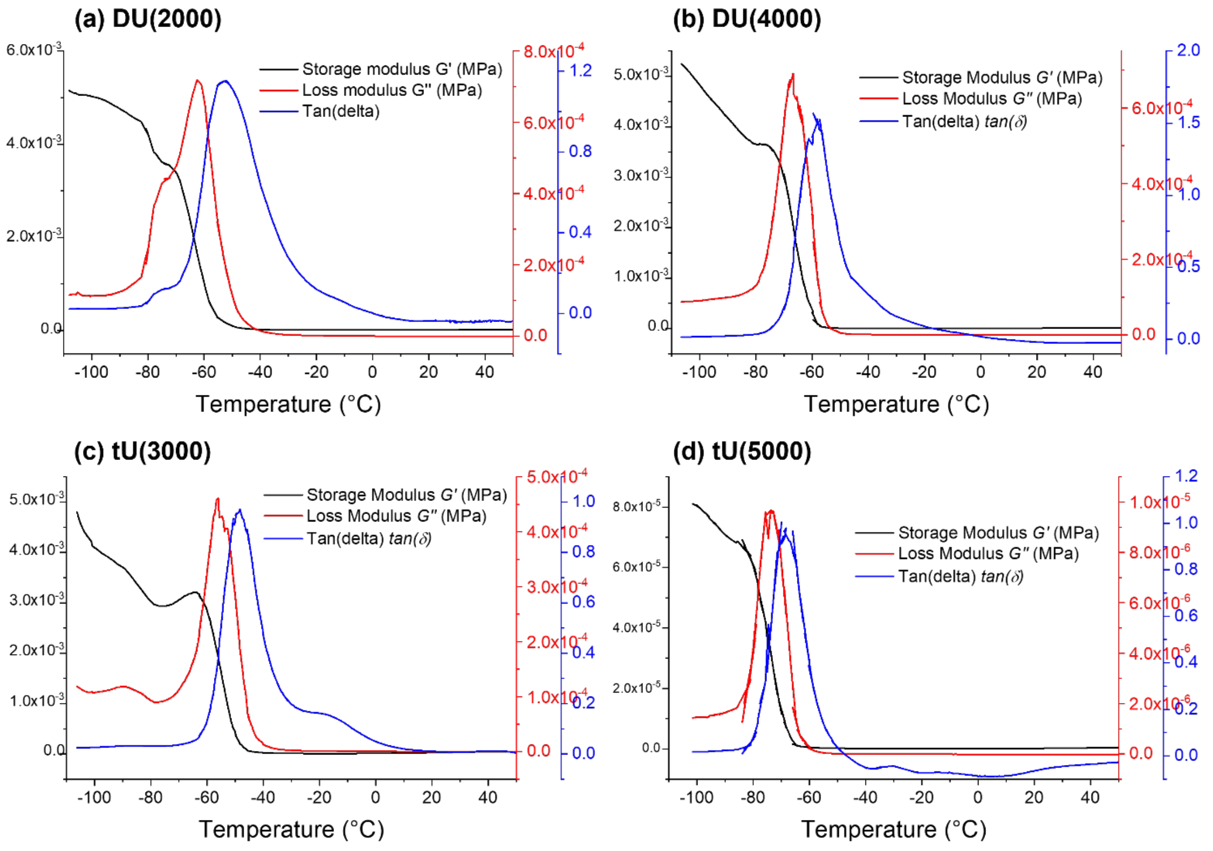

Figure S11. DMA of undoped ureasils using the 4-point bend method used from -100 to 300 °C at a rate of 5 °C/minute. Storage modulus *G*' (black lines), loss modulus *G''* (red lines) and *tan*(*δ*) (ratio of the loss to the storage, blue lines)

Table S7. Glass transition temperatures (T_g) of ureasils determined from DMA.

Ureasil	Onset T_g (°C) [a]	*G*'' Peak (°C)	Tan(*δ*) Peak (°C)
DU(2000)	-70	-62	-53
DU(4000)	-73	-70	-58
tU(3000)	-64	-56	-49
tU(5000)	-82	-75	-69

[a] Estimated glass transition temperature. Determined from calculated intersection of two tangents around the onset point of inflection of storage modulus (blank line, Figure S10).

6.2. FTIR analysis

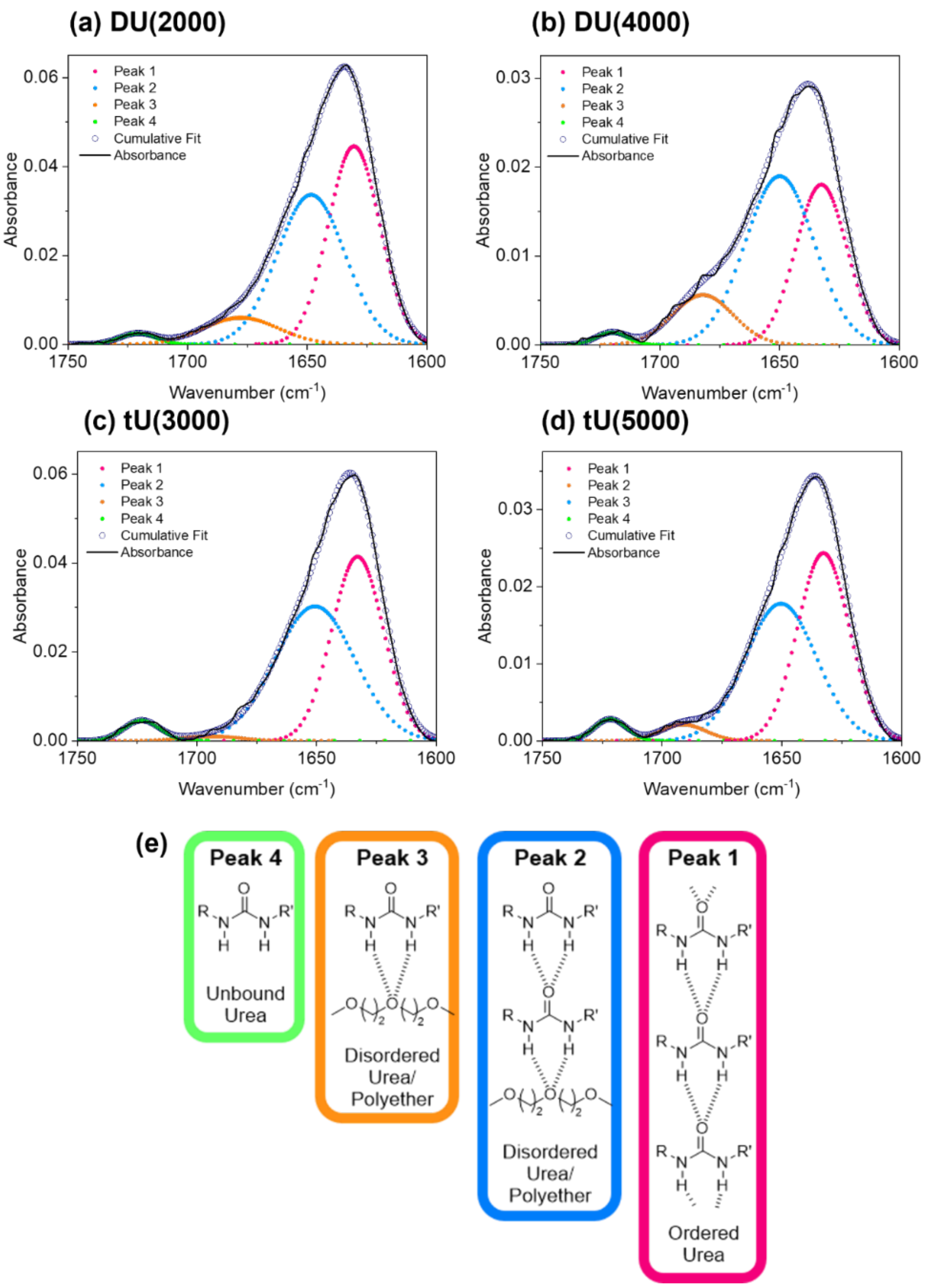

Figure S12. Gaussian deconvolution of the Amide I region in the FTIR spectra of undoped ureasils: (a) DU(2000), (b) DU(4000), (c), tU(3000), (d) tU(5000). Peaks 1-4 refer to the contributions from different hydrogen bonding environments, detailed in (e).

Table S8. Gaussian deconvolution of the Amide I region (1600-1750 cm^{-1}) in the FTIR spectra of undoped ureasils.

Sample	Peak 1			Peak 2			Peak 3			Peak 4		
	Peak centre (cm^{-1})	Integrated area	Area %	Peak centre (cm^{-1})	Integrated area	Area %	Peak centre (cm^{-1})	Integrated area	Area %	Peak centre (cm^{-1})	Integrated area	Area %
DU(2000)	1634	1	40	1655	0.39	15	1699	0.06	2.4	1724	1.08	42
DU(4000)	1632	0.48	35	1649	0.69	50	1682	0.18	13	1720	0.02	1.7
tU(3000)	1632	1.12	45	1650	1.27	51	1694	0.03	1.3	1723	0.08	3.4
tU(5000)	1633	0.66	46	1650	0.66	47	1691	0.05	3.5	1722	0.05	3.4

6.3. Mechanical characterization of ureasils

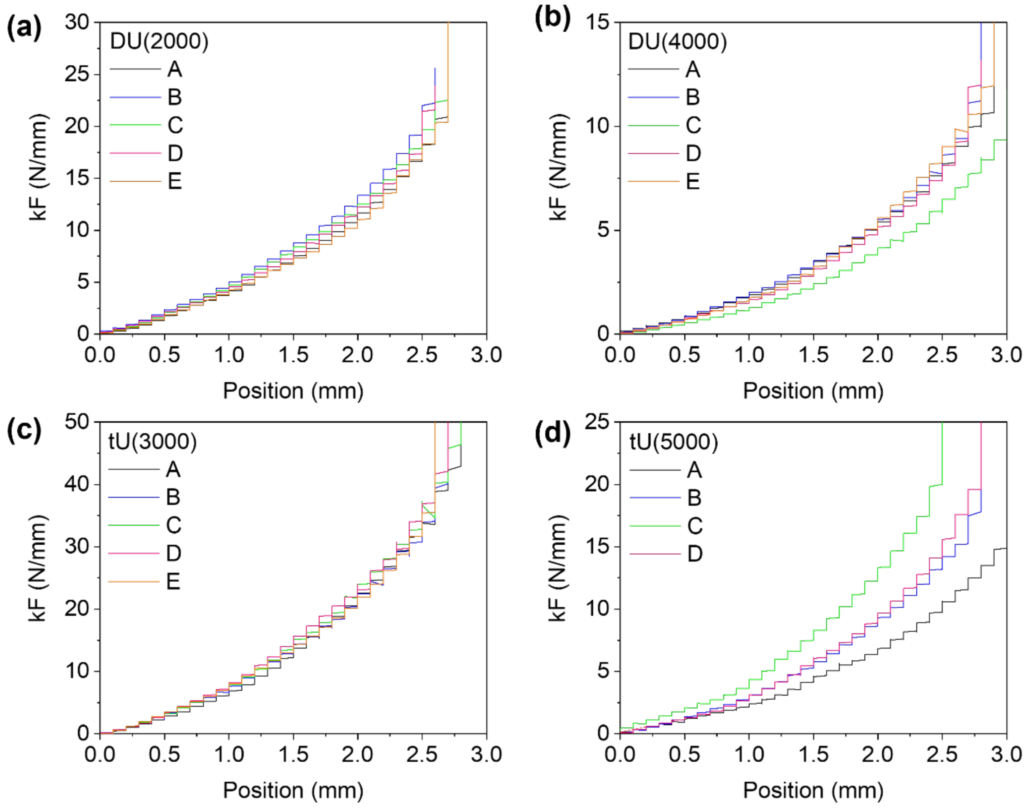

Figure S13. 4-Point bend testing data from (a) DU(2000), (b) DU(4000), (c) tU(3000) and (d) tU(5000). DU(4000) showed the lowest bending modulus, as found by a linear fit to the first 500 points of data. Labels A-E refer to datasets for repeat samples which yield an average constant and associated error. tU(3000) had the highest modulus due to higher crosslinking density and inorganic silica content. Calculated bending moduli are summarised in Table 2.

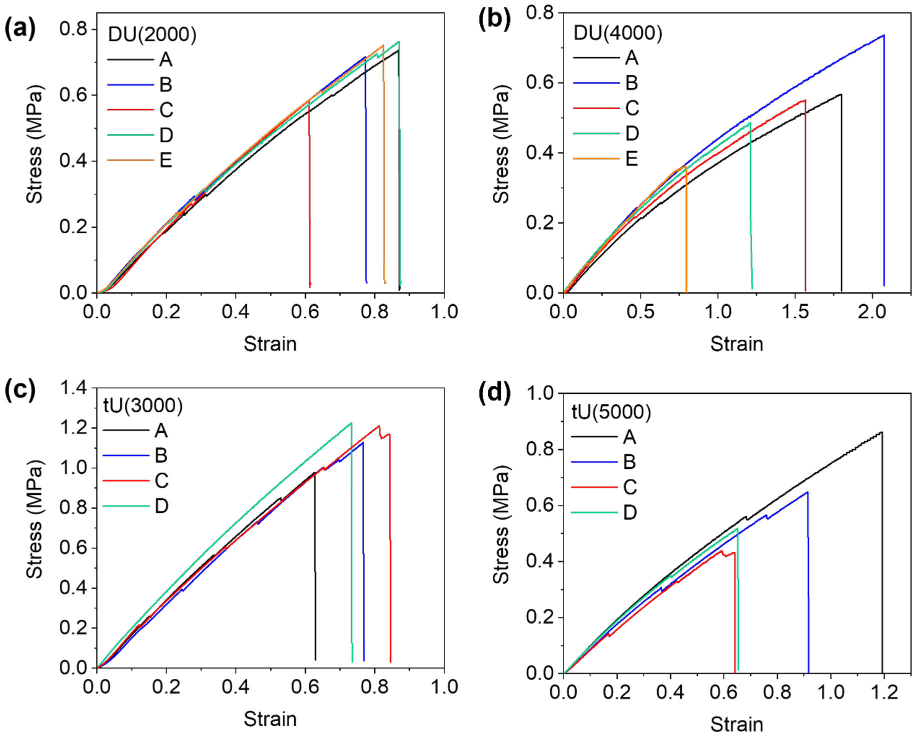

Figure S14. Tensile testing data from (a) DU(2000), (b) DU(4000), (c) tU(3000) and (d) tU(5000). Labels A-E refer to datasets for repeat samples which yield an average constant and associated error. DU(4000) showed the lowest flexural modulus, as found by a linear fit to the first 100 points of data. tU(3000) had the highest modulus due to higher crosslinking density and inorganic silica content.

6.4. PXRD: determination of structural unit distance and coherence length

Powder X-ray diffraction was used to analyze the inorganic domains in ureasil hosts. A typical ureasil diffraction pattern consists of a primary band (Band 1, Figure S15) centred at ~20°, with a shoulder at ~15-18°(Band 2, Figure S15).[10,11] Ordering within the siliceous domains gives rise to the main peak, while in-plane ordering within the siliceous domains is ascribed to the lower intensity shoulder peak.[12] The coherence length along which the structural unit survives, L, can be calculated from the primary peak using the Scherrer equation:[13]

$$L = \frac{0.94\lambda}{A\, cos\theta} \qquad \textbf{(Equation S4)}$$

where λ is the wavelength of the X-ray radiation (Cu, λ = 1.5406 nm) and A is the full-width-half-maximum of the Bragg peak in radians.

The structural unit distance, *d*, is calculated using Bragg's law:

$$2d\, sin\theta \;=\; n\lambda \qquad \textbf{(Equation S5)}$$

where *n* is a positive integer (in this case, n=1), λ is the X-ray wavelength and θ is the angle of incident radiation.

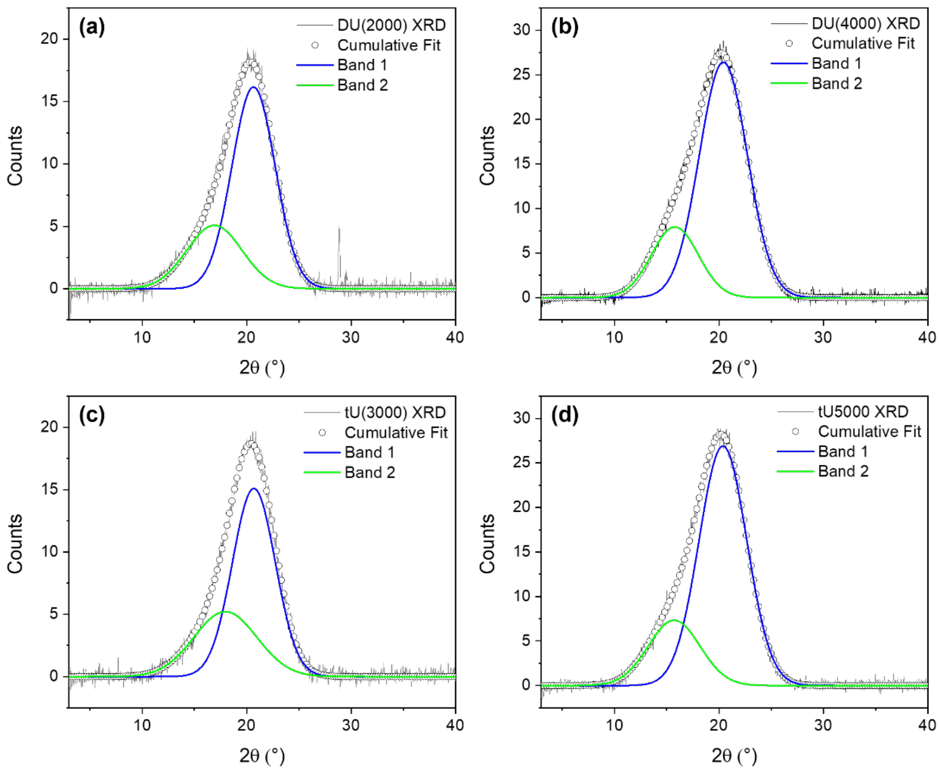

Figure S15. PXRD spectra of undoped ureasils. The original processed spectrum (black line), band 1 contribution (blue line), band 2 contribution (green line) and cumulative fit of Gaussians (white circles) are all shown.

Table S9. Gaussian deconvolution contributions of PXRD spectra and calculated silica domains.

Sample	Band 1				Band 2				*R*	X^2	Coherence length *L*, (Å)	Structural unit distance, *d* (Å)
	Peak centre (°)	FWHM[a]	Integrated area[b]	Area (%)	Peak centre (°)	FWHM[a]	Integrated area[c]	Area (%)				
DU(2000)	20.66	4.88	84.02	71.36	16.92	6.23	33.73	28.64	0.989	0.266	17.28	4.30
DU(4000)	20.42	5.42	152.36	77.33	15.79	5.29	44.66	22.67	0.996	0.219	15.55	4.34
tU(3000)	20.70	4.85	77.86	66.40	17.97	7.10	39.39	33.60	0.992	0.220	17.39	4.28
tU(5000)	20.39	5.39	154.28	77.24	15.71	5.82	45.47	22.76	0.996	0.267	15.64	4.35

[a] Full-width at half-maximum [b] Range = 10-25° [c] Range = 13-30°

6.5. Comparison of ambient and degassed DPA/PdOEP doped tU(3000)

Table S10. Key photophysical parameters of tU(3000) doped with 10 mM DPA and 0.1 mM PdOEP, when prepared in either entirely ambient conditions, or when the doped t-UPTES solution was degassed with nitrogen bubbling for one hour prior to gelation in ambient conditions.

	tU(3000) host ambient	tU(3000) host degassed
Integrated emission intensity (counts) at 100 mW cm^{-2}	1.50 x 10^5	6.44 x 10^5
Φ_{UC} at 100 mW cm^{-2}	0.42 ± 0.04%	0.39%
Integrated emission intensity (counts) at 1 W cm^{-2}	8.88 x 10^6	1.75 x 10^7
Φ_{UC} at 1 W cm^{-2}	1.65 ± 0.20%	2.39 ± 0.30%
$<\tau_{UC}>^a$	22 ± 2.5 ms	24.9 ms
$<\tau_{PHOS}>^b$	59 µs	289 µs

532 nm laser excitation was used for all above measurements. [a] Average upconversion lifetime (λ_{ex} = 532 nm, λ_{em} = 440 nm). [b] Average phosphorescence lifetime in presence of DPA (λ_{ex} = 532 nm, λ_{em} = 665 nm). All measurements were performed under ambient conditions. Errors are the standard deviation of two measurements.

7. References

(1) Porrès, L.; Holland, A.; Pålsson, L. O.; Monkman, A. P.; Kemp, C.; Beeby, A. Absolute Measurements of Photoluminescence Quantum Yields of Solutions Using an Integrating Sphere. *J. Fluoresc.* **2006**, *16* (2), 267–273. https://doi.org/10.1007/S10895-005-0054-8.

(2) Ahn, T. S.; Al-Kaysi, R. O.; Müller, A. M.; Wentz, K. M.; Bardeen, C. J. Self-Absorption Correction for Solid-State Photoluminescence Quantum Yields Obtained from Integrating Sphere Measurements. *Rev. Sci. Instrum.* **2007**, *78* (8), 086105. https://doi.org/10.1063/1.2768926.

(3) De Mello, J. C.; Wittmann, H. F.; Friend, R. H. An Improved Experimental Determination of External Photoluminescence Quantum Efficiency. *Adv. Mater.* **1997**, *9* (3), 230–232. https://doi.org/10.1002/adma.19970090308.

(4) Zhou, Y.; Castellano, F. N.; Schmidt, T. W.; Hanson, K. On the Quantum Yield of Photon Upconversion via Triplet–Triplet Annihilation. *ACS Energy Lett.* **2020**, *5* (7), 2322–2326. https://doi.org/10.1021/acsenergylett.0c01150.

(5) Lakowicz, J. R. *Principles of Fluorescence Spectroscopy*; Springer, 2006. https://doi.org/10.1007/978-0-387-46312-4.

(6) Collins, A.; Southern, T.; Lyu, G.; Bennison, M. J.; Evans, R. C. Photoactive Organic-Inorganic Hybrid Polymer Waveguides for Optical Device Technologies. In *Photosensitive Materials and their Applications*; McLeod, R. R., Tomita, Y., Sheridan, J. T., Pascual Villalobos, I., Eds.; SPIE, 2020; Vol. 11367, p 32. https://doi.org/10.1117/12.2564510.

(7) Carlos, L. D.; Sá Ferreira, R. A.; Pereira, R. N.; Assunção, M.; De Zea Bermudez, V. White-Light Emission of Amine-Functionalized Organic/Inorganic Hybrids: Emitting Centers and Recombination Mechanisms. *J. Phys. Chem. B* **2004**, *108* (39), 14924–14932. https://doi.org/10.1021/jp049052r.

(8) Bachilo, S. M.; Weisman, R. B. Determination of Triplet Quantum Yields from Triplet-Triplet Annihilation Fluorescence. *J. Phys. Chem. A* **2000**, *104* (33), 7713–7714. https://doi.org/10.1021/jp001877n.

(9) Chen, Q.; Liu, Y.; Guo, X.; Peng, J.; Garakyaraghi, S.; Papa, C. M.; Castellano, F. N.; Zhao, D.; Ma, Y. Energy Transfer Dynamics in Triplet-Triplet Annihilation Upconversion Using a Bichromophoric Heavy-Atom-Free Sensitizer. *J. Phys. Chem. A* **2018**, *122* (33), 6673–6682. https://doi.org/10.1021/acs.jpca.8b05901.

(10) Meazzini, I.; Willis-Fox, N.; Blayo, C.; Arlt, J.; Clément, S.; Evans, R. C. Targeted Design Leads to Tunable Photoluminescence from Perylene Dicarboxdiimide-Poly(Oxyalkylene)/Siloxane Hybrids for Luminescent Solar Concentrators. *J. Mater. Chem. C* **2016**, *4* (18), 4049–4059. https://doi.org/10.1039/c5tc03952e.

(11) Fu, L.; Sá Ferreira, R. A.; Silva, N. J. O.; Carlos, L. D.; De Zea Bermudez, V.; Rocha, J. Photoluminescence and Quantum Yields of Urea and Urethane Cross-Linked Nanohybrids Derived from Carboxylic Acid Solvolysis. *Chem. Mater.* **2004**, *16* (8), 1507–1516. https://doi.org/10.1021/cm035028z.

(12) Lima, P. P.; Ferreira, R. A. S.; Júnior, S. A.; Malta, O. L.; Carlos, L. D. Terbium(III)-Containing Organic-Inorganic Hybrids Synthesized through Hydrochloric Acid Catalysis. *J. Photochem. Photobiol. A Chem.* **2009**, *201* (2–3), 214–221. https://doi.org/10.1016/j.jphotochem.2008.10.021.

(13) Borie, B. X-Ray Diffraction in Crystals, Imperfect Crystals, and Amorphous Bodies. *J. Am. Chem. Soc.* **1965**, *87* (1), 140–141. https://doi.org/10.1021/ja01079a041.
